# The Functional Impact of Mitochondrial Structure Across Subcellular Scales

**DOI:** 10.3389/fphys.2020.541040

**Published:** 2020-11-11

**Authors:** Brian Glancy, Yuho Kim, Prasanna Katti, T. Bradley Willingham

**Affiliations:** ^1^Muscle Energetics Laboratory, NHLBI, National Institutes of Health, Bethesda, MD, United States; ^2^NIAMS, National Institutes of Health, Bethesda, MD, United States; ^3^Department of Physical Therapy and Kinesiology, University of Massachusetts Lowell, Lowell, MA, United States

**Keywords:** mitochondria, energetics, cristae, mitochondrial dynamics, mitochondrial networks, organelle interaction

## Abstract

Mitochondria are key determinants of cellular health. However, the functional role of mitochondria varies from cell to cell depending on the relative demands for energy distribution, metabolite biosynthesis, and/or signaling. In order to support the specific needs of different cell types, mitochondrial functional capacity can be optimized in part by modulating mitochondrial structure across several different spatial scales. Here we discuss the functional implications of altering mitochondrial structure with an emphasis on the physiological trade-offs associated with different mitochondrial configurations. Within a mitochondrion, increasing the amount of cristae in the inner membrane improves capacity for energy conversion and free radical-mediated signaling but may come at the expense of matrix space where enzymes critical for metabolite biosynthesis and signaling reside. Electrically isolating individual cristae could provide a protective mechanism to limit the spread of dysfunction within a mitochondrion but may also slow the response time to an increase in cellular energy demand. For individual mitochondria, those with relatively greater surface areas can facilitate interactions with the cytosol or other organelles but may be more costly to remove through mitophagy due to the need for larger phagophore membranes. At the network scale, a large, stable mitochondrial reticulum can provide a structural pathway for energy distribution and communication across long distances yet also enable rapid spreading of localized dysfunction. Highly dynamic mitochondrial networks allow for frequent content mixing and communication but require constant cellular remodeling to accommodate the movement of mitochondria. The formation of contact sites between mitochondria and several other organelles provides a mechanism for specialized communication and direct content transfer between organelles. However, increasing the number of contact sites between mitochondria and any given organelle reduces the mitochondrial surface area available for contact sites with other organelles as well as for metabolite exchange with cytosol. Though the precise mechanisms guiding the coordinated multi-scale mitochondrial configurations observed in different cell types have yet to be elucidated, it is clear that mitochondrial structure is tailored at every level to optimize mitochondrial function to meet specific cellular demands.

## Introduction

Mitochondria are no longer known simply as the powerhouse of the cell as they have now been shown to play a key part in several cellular processes including metabolite biosynthesis and signaling beyond their classical role in energy metabolism. However, the relative mitochondrial contribution to each of these different cellular processes is determined by the specific demands placed on the cell. For example, the primary function of striated muscle cells is to generate force through muscle contraction. As a result, the main role of mitochondria in striated muscle cells is to convert fuel into the energy needed to sustain this relatively high energy demand task. In contrast, the liver plays a much larger role in the synthesis of proteins, carbohydrates, and lipids, among other products. In support of this increased cellular synthesis demand, the mitochondria in the liver have a relatively greater capacity for metabolite biosynthesis and lower energy conversion capabilities compared to striated muscle mitochondria (Johnson et al., 2007; Phillips et al., 2012). While it is becoming increasingly well established that mitochondrial protein composition can vary largely among different cell types (Mootha et al., 2003; Johnson et al., 2007; Pagliarini et al., 2008; Calvo et al., 2016; Benador et al., 2018), how mitochondrial structural alterations contribute to the wide range of mitochondrial functional capacities observed in different cells is less well understood. Therefore, the aim of this review is to discuss current knowledge of how mitochondrial structure can be modulated and coordinated across different spatial scales to support cells facing diverse functional demands. Highlighting how mitochondrial structure is tightly regulated within a cell, we focus on the functional trade-offs associated with structural alterations within a mitochondrion, at the single organelle and mitochondrial network levels, and between mitochondria and other organelles which contribute to cellular functional specificity.

## Internal Mitochondrial Structure

**FIGURE 1:**
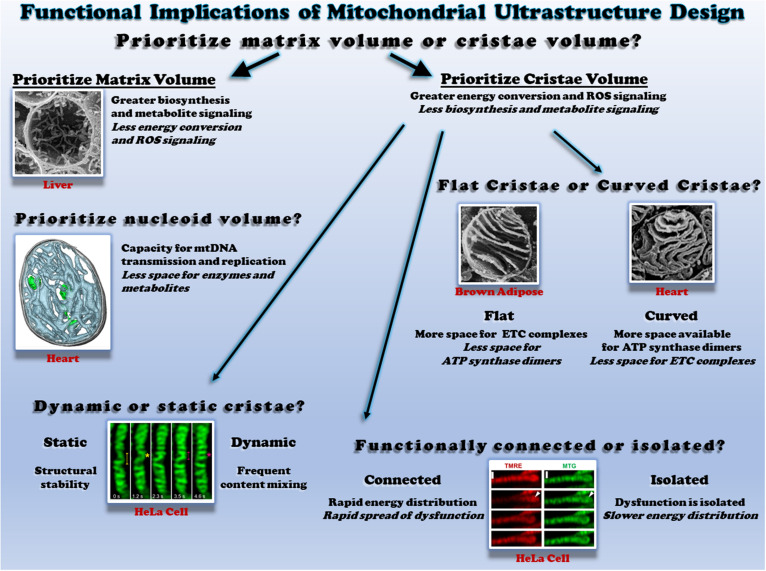
Functional consequences of different mitochondrial ultrastructure configurations. Top: the relative proportion of mitochondrial volume occupied by cristae or the matrix dictates space available to perform different functions. Scanning electron micrograph (SEM) of a liver mitochondrion from (Bochimoto et al., 2017). Middle right: the relative proportion of flat and curved cristae regions determines the space available for different oxidative phosphorylation enzymes. SEM of brown adipose tissue (Cinti, 2018) and heart mitochondria (Kanzaki et al., 2010) on the left and right, respectively. Middle left: mitochondrial nucleoids share the matrix space with enzymes and metabolites. Cryo-electron tomogram of a heart mitochondrion showing cristae (cyan) and nucleoids (green) from (Kanzaki et al., 2010; Kukat et al., 2015). Bottom left: the rate of cristae dynamics determines the frequency of remodeling and content sharing. Cristae remodeling in HeLa cells shown from (Kondadi et al., 2020). Bottom right: the relative electrical connectivity of cristae regulates distribution rate. Depolarization of some but not all HeLa cell cristae from (Wolf et al., 2019). All figures reproduced with permission.

Within the mitochondrial outer membrane lies a tortuous mitochondrial inner membrane which can be further separated into an inner boundary membrane running parallel to the outer membrane and the cristae folds protruding into the mitochondrial matrix (Harmon et al., 1974; Frey and Mannella, 2000; Mannella et al., 2013; Cogliati et al., 2016; Tobias et al., 2018). The cristae, rather than the inner boundary membrane, are reported to contain the majority of electron transport chain (ETC) complexes and ATP synthase dimers which, respectively, generate and consume the protonmotive force across the membrane to produce ATP (Gilkerson et al., 2003; Davies et al., 2012; Wilkens et al., 2013; Cogliati et al., 2016). Thus, mitochondria with relatively more cristae are likely to have greater capacities for energy conversion compared with those with less cristae (Figure 1, top). The ETC complexes are also the primary mitochondrial site of reactive oxygen species (ROS) production (Chandel et al., 2000; Chandel, 2014; Brand, 2016; Wong et al., 2017), implying that cristae membrane volume may also determine capacity for ROS signaling. The trade-off associated with increasing the relative mitochondrial volume occupied by cristae, however, is a concomitant reduction in mitochondrial matrix volume. This necessary volumetric exchange has been well described, for example, during postnatal development in both the heart and liver (Smith and Page, 1977; Kanamura et al., 1985). In the rabbit heart three days before birth, the matrix makes up 48% of mitochondrial volume while the inner boundary membrane + cristae take up 39%. However, two days after birth, the transition to more aerobic metabolism results in a redistribution of mitochondrial volume where the inner membrane + cristae occupy more volume than the matrix (47 vs. 42%, respectively) (Smith and Page, 1977). The postnatal transition is slightly altered in the liver and also depends on which region of the liver is assessed; relative matrix volume decreases from 81% to 75% throughout maturation in the perihepatic regions whereas matrix volume decreases from 81% to 75% in the five days after birth in the periportal regions but returns back to 81% by adulthood (Kanamura et al., 1985). Note also that the liver matrix volume is nearly twice that of the heart in these two studies. While acutely condensing the mitochondrial matrix space in response to an energetic challenge would increase matrix metabolite concentrations and propel the energy conversion process temporarily (Hackenbrock, 1966), long term reduction of matrix space means less room available to be occupied by matrix enzymes and mitochondrial DNA nucleoids, suggesting lower capacity for mitochondrial matrix functions as well as mitochondrial DNA transmission and replication. Metabolites generated by the tricarboxylic acid (TCA) cycle in the matrix have recently been identified as important regulators of gene activity (Chandel, 2014; Martínez-Reyes and Chandel, 2020), and the matrix is home to key reactions within several essential biosynthesis pathways often involving extramitochondrial components including purine nucleotide, amino acids, glucose, heme, urea, fatty acids, and cholesterol synthesis (Frezza, 2017; Spinelli and Haigis, 2018). Thus, in cells with greater demand for biosynthesis and signaling, maintaining a larger matrix relative to cristae volume may be advantageous. Indeed, the larger matrix volume in the liver relative to the heart mentioned above is consistent with that liver mitochondria contain less ETC protein content and activity compared to the heart across several species (Phillips et al., 2012), reflecting the relative demands for synthesis and energy conversion among these cell types and demonstrating the close relationship between mitochondrial ultrastructure and function.

### Cristae Shape

Individual cristae can take several different shapes which are regulated in part by the mitochondrial contact site and cristae organizing system (MICOS) and Opa1 (Olichon et al., 2002; Frezza et al., 2006; Harner et al., 2011; Kondadi et al., 2020). The structural differences observed between lamellar cristae which are more disk or sheet-like in nature and tubular cristae which are more cylindrical may offer distinct functional advantages and disadvantages (Figure 1, middle right). As mentioned above, ETC complexes and ATP synthase dimers are each located primarily within the cristae. However, electron cryotomography studies have suggested that ATP synthase dimers are preferentially located in and perhaps even help form the more curved regions of the cristae such as the tips (Davies et al., 2012; Mühleip et al., 2016), whereas the ETC complexes are located in less curved regions such as the stalks (Vogel et al., 2006; Ikon and Ryan, 2017). These results suggest that the relative curvature of the cristae may regulate the ratio of ETC complexes to ATP synthase within a mitochondrion. Indeed, mitochondria from brown adipose tissue, which are well known for high energy dissipation capacity through uncoupling, have lamellar cristae which appear to extend across the entire width of the mitochondrion (Lever and Chappell, 1958; Inoué and Koike, 1989; Perkins et al., 1998). By extending across the entire organelle and minimizing the curved regions of the cristae, brown fat mitochondria thus have less available space to place ATP synthase dimers, while still maintaining a large region for placement of ETC complexes. In contrast, in heart and skeletal muscle mitochondria where energetic efficiency is critical to sustaining muscle contraction, the lamellar cristae display much more curvature than in brown adipose tissue despite similar cristae densities (Kang et al., 2009; Picard et al., 2015; de-Lima-Junior et al., 2019). In each case, cristae structure appears to be tuned to support the energetic needs of the respective cells.

### Cristae Dynamics

Investigations into mitochondrial cristae morphology have traditionally required the use of electron microscopy on fixed specimens to achieve the resolution necessary to observe these structures which are often 30 nm or less in diameter (Palade, 1953; Frey and Mannella, 2000; Mannella, 2006; Hoppel et al., 2009; Zick et al., 2009; Picard et al., 2015). As a result, cristae have traditionally been thought of as stable structures despite the widespread observations of individual mitochondria as dynamic structures over the past two decades (Nunnari et al., 1997; Bleazard et al., 1999; Karbowski et al., 2004; Szabadkai et al., 2004; Twig et al., 2008b; Molina et al., 2009; Lewis et al., 2016). However, recent technological advances have brought the resolution of the light microscope down into the tens of nanometer range which has finally permitted observation of cristae structures in live cells (Dlasková et al., 2018, 2019; Ježek and Dlasková, 2019; Jakobs et al., 2020; Figure 1 bottom left). Application of these super-resolution microscopy techniques to mitochondria from several types of cell culture systems have indeed revealed that cristae are dynamic structures capable of both fission and fusion-type events similar to those which occur at the organelle level (Huang et al., 2018; Stephan et al., 2019; Wang et al., 2019). Earlier this year, cristae dynamics in cultured HeLa and HEK293T cells were shown to be regulated by MICOS proteins MIC13, which was required for both cristae junction and cristae membrane dynamics, and MIC60, which appeared to act as a docking site for cristae junctions (Kondadi et al., 2020). Further, cristae dynamics events were classified in this work as either transverse, Y-type, and X-type where the events occurred perpendicular to the mitochondrial short axis, or through transient Y or X-shaped structures, respectively (see Figure 9 in Kondadi et al., 2020 for examples). As investigations into cristae dynamics are only in their infant stages at present, the functional significance of these events has yet to be tested in physiological systems. It remains to be understood how the frequency of cristae dynamics is modulated across differentiated cell types, in response to different stimuli or under various pathological conditions. If the range of cristae dynamics frequencies across cell types and physiological conditions is parallel to that of individual mitochondrial dynamics, there may be some mitochondria with highly dynamic cristae like those observed thus far in cell culture, while other mitochondria may have more stable cristae structures similar to the relatively low mitochondrial dynamics rates reported in mature striated muscles (Eisner et al., 2014, 2017). Gaining a better understanding of how remodeling cristae shapes through these dynamics events may affect the placement and function of ETC complexes as well as the ATP synthase would also provide keen insight into how the structural changes associated with cristae dynamics are related directly to mitochondrial function.

### Cristae Energetics

It has long been assumed that the protonmotive force generated across the mitochondrial inner membrane is uniform throughout the entire organelle thereby making a single mitochondrion akin to a single cell battery. Indeed, it has been proposed that elongated mitochondria can act as a power cable to distribute the electrical component of the protonmotive force, the membrane potential, across long cellular distances (Skulachev, 1969, 1977, 1990; Patel et al., 2016). In support of this power cable hypothesis, localized depolarization of mitochondria within a cell has been shown to result in rapid depolarization of mitochondria over 10 μm away in cultured fibroblasts and neonatal cardiomyocytes (Amchenkova et al., 1988), and more recently in freshly isolated skeletal and cardiac myocytes from mice (Glancy et al., 2015, 2017; Bleck et al., 2018). However, owing again to the advancements in super-resolution microscopy allowing visualization of cristae structures in live cells, recent data suggest that it is also possible for individual cristae to be electrically isolated within a mitochondrion (Wolf et al., 2019). Using fluorescent lipophilic cation dyes which accumulate in proportion to the mitochondrial membrane potential in a variety of cultured cell types, it was reported that individual cristae can maintain different membrane potentials within a single mitochondrion and that the cristae maintain a higher membrane potential than the inner boundary membrane in a MICOS and Opa1 dependent manner. Further, the functional consequences of electrical isolation of individual cristae were tested by depolarizing one end of a single, elongated fibroblast mitochondrion and observing whether the remainder of the mitochondrion depolarized instantaneously (within 0.15 s) or in a wave-like manner. Indeed, the majority of the mitochondria depolarized in a wave-like manner as would be predicted if cristae were functionally independent. However, it is important to note that 20% of the mitochondria tested in this study depolarized instantaneously which supports the single cell battery model of mitochondrial function. These mixed results coupled together with the similarly instantaneous mitochondrial depolarizations observed in striated muscle mitochondria (Glancy et al., 2015, 2017; Bleck et al., 2018) suggest that electrical isolation of individual cristae is the result of one possible structural configuration, whereas an electrically united mitochondrion is another possible configuration (Figure 1, bottom right). The full functional consequences of maintaining cristae as energetically independent units are not yet understood though electrical isolation would inherently slow the spread of any potential dysfunction and provide more time to mount a protective response. On the other hand, as shown with the above mentioned wave-like depolarizations down the length of a mitochondrion, electrically isolating the cristae delays the distribution of membrane potential electrical energy to regions where cellular work has suddenly increased thereby resulting in greater ATP supply and demand mismatches as well as relatively lower capacity for membrane potential-dependent metabolites (e.g., glutamate/aspartate) and ion (e.g., Ca^2+^) exchange. Thus, it may be advantageous for a relatively large cell which faces rapid changes in energy demand, such as the skeletal muscle, to maintain a uniform membrane potential throughout the entire mitochondrion in order to more optimally respond to cellular energy demands.

## Individual Mitochondrial Structure

In a cell, mitochondrial shapes and dynamics are finely tuned by fusion and fission proteins whose molecular actions are dependent on the presence of GTP. Mitochondrial elongation is initiated by a gradual merge of the outer mitochondrial membranes (OMM) of two individual mitochondria, for which mitofusin (Mfn) proteins, Mfn1 and Mfn2, are required. Afterward, the inner mitochondrial membranes (IMM) are fully joined by the action of OPA1, and the cristae from separate mitochondria are formed into a new crista structure wherein mtDNA, ions, and other small molecules can be shared. On the contrary, a single elongated mitochondrion can be fragmented into smaller structures through recruiting a group of proteins including a dynamin-like protein Drp1 and its associated proteins such as Mff, Fis1, and Mid49/51. For detailed molecular mechanisms behind mitochondrial fusion and fission systems, see (Detmer and Chan, 2007; Westermann, 2010; Friedman and Nunnari, 2014; Chan, 2019; Dorn, 2019). In addition to modulating mitochondrial structure, these mitochondrial dynamics proteins also play a critical role in maintaining mitochondrial quality control (Twig et al., 2008b). It is often difficult to separate the effects of altering the quality control systems from the effects of alterations in shape, and therefore, the functional changes associated with manipulations of mitochondrial dynamics proteins discussed below may indeed be more related to modulating the mitochondrial quality control systems than to specific changes in individual mitochondrial shape.

Mitochondrial fusion-mediated elongation is accomplished by the cooperative action of outer (e.g., Mfn1/2) and inner (e.g., OPA1) membrane machineries, by which mitochondrial function and homeostasis are maintained (Chan, 2012; Youle and van der Bliek, 2012). Mitochondrial fusion contributes to establish mtDNA stability, as lack of fusion proteins (e.g., double-deletion of Mfn1/2) significantly impaired mtDNA quantity and quality controls in skeletal muscle (Chen et al., 2010). Recently, it was also shown that mitochondrial fusion is important for the regulation of both mtDNA replication machinery and mtDNA nucleoid distribution (Ramos et al., 2019). In the Mfn-defective muscles, mitochondrial oxygen consumption and ATP production were notably downregulated (Chen et al., 2010), indirectly suggesting a connection between mitochondrial structure and oxidative function. In addition, it has been identified that OPA1 associated-mitochondrial tubulation is important for mitochondrial quality control (Ishihara et al., 2006; Quintana-Cabrera et al., 2018). Upon complex III inhibition, for example, overexpression of OPA1 protects mitochondrial oxidative capacity through stabilizing ATP synthase function (Quintana-Cabrera et al., 2018).

Mitochondrial fission plays a significant role in both mitochondrial oxidative function (Favaro et al., 2019) and initial apoptotic signaling (Youle and Karbowski, 2005). Dysregulation of Drp1, a key player for the mitochondrial fission machinery, was shown to lead to elongated mitochondria, which appeared to enhance cellular resistance against the induction of cell death signaling (Cribbs and Strack, 2007). Given that Drp1-associated mitochondrial segmentation is dependent on the action of calcineurin, a protein phosphatase 3 (Cribbs and Strack, 2007), several studies have explored possible linkages between mitochondrial dynamics and calcium handling. For example, in the Drp1-deleted mouse muscle, it was observed that an electrical stimulation-induced calcium response was largely diminished, while swollen mitochondria showed increased calcium uptake along with upregulation of the mitochondrial calcium uniporter (MCU) (Favaro et al., 2019). In addition to skeletal muscle, mitochondrial fission also affects cellular and physiological functions in other tissues. For example, beta cell-specific deletion of Drp1 resulted in not only impaired mitochondrial morphology but also abnormal insulin secretion, although oxygen consumption rate was not significantly affected (Hennings et al., 2018).

The mitochondrial dynamics system is also known for regulating the response to various physiological challenges. Upon starvation, mitochondria form tubular, elongated structures, which have been identified to protect cells from autophagosomal degradation (Gomes et al., 2011) and promote cristae remodeling, mtDNA sharing, and delayed apoptotic signaling (Rambold et al., 2011). These mitochondrial elongation-associated quality controls are dependent on fusion machinery. For example, in mitochondrial fusion impaired cells (i.e., OPA1 KO MEFs), mitochondrial protein abundances for Tom20, Hsp60, and Complex V are significantly down-regulated following serum starvation (Rambold et al., 2011). On the other hand, overnutrition such as hyperglycemia is linked not only with mitochondrial fragmentation (Yu et al., 2019), but also with mitochondrial dysfunction (Bonnard et al., 2008). A hyperglycemia-associated mitochondrial shape transition occurs along with ROS production, which is reversible with a promotion of mitochondrial fusion proteins such as Mfn2 (Yu et al., 2006). Meanwhile, calcineurin-dependent Drp1 dephosphorylation has been shown to move the balance of mitochondrial dynamics toward greater fission (Cereghetti et al., 2008). In the calcineurin-ablated skeletal muscle of mice, elongated mitochondria are more prevalent and they are protected from high-fat diet-associated mitochondrial dysregulation, suggesting an essential role of calcineurin-dependent mitochondrial remodeling upon nutritional challenges (Pfluger et al., 2015).

Mitochondrial morphology and function are both notably altered during cellular development and senescence. Recently, it was shown that proliferating cells accomplish increased mitochondrial oxidative capacity not by mitochondrial biogenesis but by mitochondrial elongation (Yao et al., 2019). As compared to quiescent cells whose morphology is relatively short and fragmented, proliferating cells have more tubular mitochondria, as well as an upregulation of mitochondrial fusion protein abundance (Yao et al., 2019). On the other hand, cellular aging has been also characterized by mitochondrial elongation, although the exact underlying mechanism is not clear yet. In skeletal muscle of old animals, mitochondria located in intermyofibrillar structures are more elongated and branched compared to younger muscles due to an increase in mitochondrial fusion (Leduc-Gaudet et al., 2015). Furthermore, it has been well documented that aging is often associated with reduced mitochondrial function and vitality, and aged tissues including skeletal muscles have severely impaired mitochondrial energetics, biogenesis, and quality controls, as well as imbalanced oxidative stress (Shigenaga et al., 1994; Lopez-Otin et al., 2013).

In addition to aging, pathological conditions have also shown significantly altered mitochondrial dynamics and morphology, as well as mitochondrial dysfunction (Tsushima et al., 2018). For instance, it has been well documented that dysregulated mitochondrial fusion plays a critical role for Charcot-Marie Tooth disease type 2 A, the inherited neurodegenerative disease (Franco et al., 2016; Zhou et al., 2019). Huntington’s disease, another neurological disorder, is also characterized by highly upregulated Drp1-associated fission, as well as fragmented mitochondrial formation (Shirendeb et al., 2012). Meanwhile, given that mitochondrial shapes in cancer cells are more fragmented and associated with poor mitochondrial capacity (Anderson et al., 2018), cancer treatments have been targeted to fix mitochondrial dynamics and dysfunction through promoting mitochondrial elongation (Anderson et al., 2018; Yu et al., 2019). Hence, the irregular mitochondrial shapes and functions observed in various pathological settings have been recognized as a key leading factor for various diseases, as well as a target system for the pharmaceutical treatment, but more research will be needed to clearly define causal relationships between disease-associated mitochondrial remodeling and pathological symptoms.

Additionally, it is important to note that mitochondrial shape can also be regulated independently of mitochondrial fission/fusion proteins. Dysfunctional mitochondria have been shown to be disconnected from the mitochondrial networks found in skeletal muscle cells in a manner inconsistent with mitochondrial fission (Glancy et al., 2017). Localized depolarization caused elongated, branching mitochondria to become more condensed and spherical in nature, although the number of mitochondria decreased and the size of mitochondria increased, respectively. These results may be related to the Miro1 dependent mitochondrial shape transition also recently described where, upon cytosolic calcium stress, tubular mitochondrial structures became more fragmented independent of the mitochondrial fission process (Nemani et al., 2018).

### Irregular Mitochondrial Shapes

Several studies have displayed “ring” or “donut”-like mitochondrial structures in different cell types, which seems to be associated with mitochondrial oxidative stress (Liu and Hajnoczky, 2011; Ahmad et al., 2013). For instance, rotenone (complex I inhibitor)- and antimycin (complex III inhibitor)-induced ROS resulted in a mitochondrial structural transition from tubular to donut- or blob-shaped formation, and the ROS-associated mitochondrial donut formations were found to be transient and reversible (Ahmad et al., 2013). Nevertheless, using a combined imaging technique with light and electron microscopy, it was suggest that in response to a loss of membrane potential, many ring-shaped mitochondria did not have “true through holes” but instead had “vase-shaped cavities” (Miyazono et al., 2018). Meanwhile, donut-shaped mitochondria were recently observed by 3D electron microscopy in skeletal muscle of healthy adult mice (Bleck et al., 2018), showing that glycolytic muscle fibers have more donut-like mitochondrial structures as compared to oxidative muscle fibers. Additionally, the glycolytic muscle mitochondrial donut holes were found to be filled with sarcoplasmic reticulum and cytosol, while the holes in oxidative fibers were filled with lipid droplets, suggesting a yet to be defined functional role for mitochondrial donuts (Bleck et al., 2018). Although it is still unclear how and why mitochondria form this specific shape, it has been suggested that donut size is critical for adjusting the bending energy that is a main barrier for mitochondrial donut formations and could be counterbalanced with stress-inducible mitochondrial osmotic pressure (Long et al., 2015).

Two or more individual mitochondria can communicate with each other via thin, tubular connections called mitochondrial nanotunnels, although free-ended extruding mitochondrial nanotunnels are also observed (Vincent et al., 2017). With mitochondrial matrix specific-photoactivable fluorescent proteins (e.g., mtPA-GFP), it was shown that the tube-like structures projected from a mitochondrion can be connected with distant (∼8 μm) mitochondria in matured cardiomyocytes (Huang et al., 2013). Several studies have sought to understand the underlying mechanisms linked with the incidence of mitochondrial nanotunneling formations. Mitochondrial nanotunnels spanning at various distance (i.e., ∼1 to over 5 μm) have been shown to contribute to the transfer of mitochondrial matrix contents and are dependent on SR-mediated calcium signaling (Lavorato et al., 2017). In a study based on 3D electron tomograms, it was proposed that these nanotunnels may be formed by pulling and/or extending actions by microtubules, as many nanotunnels are arranged along with microtubules in close distance and they also have parallel cristae orientation (Lavorato et al., 2017). Also, it was shown that Miro2, a major protein for mitochondrial trafficking along the microtubules, is required in the mitochondrial nanotunneling formations in cardiomyocytes (Cao et al., 2019). Additionally, more prevalent mitochondrial nanotunneling structures were observed in skeletal muscles of mitochondrial disease patients (Vincent et al., 2019).

### Individual Mitochondrial Structure and Function Across Different Tissues

**FIGURE 2:**
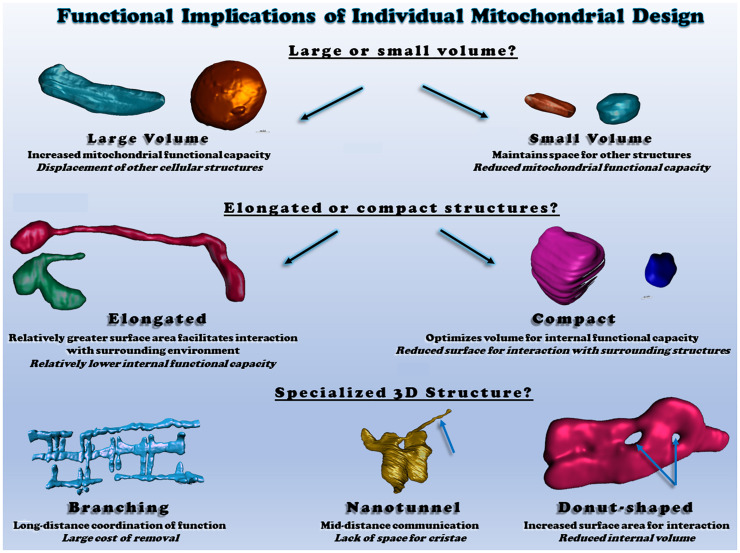
Functional consequences of different individual mitochondrial structures. Top: individual mitochondrial volume determines the relative capacity for function but displaces other structures. Middle: Elongated or compact mitochondrial shapes determine the surface area available for interactions relative to mitochondrial volume. Bottom: irregular mitochondrial shapes allow for communication and interaction across various cellular distances. All heart and skeletal muscle mitochondrial 3D renderings shown were created from raw data available within (Glancy et al., 2017; Bleck et al., 2018).

Tissue-specific mitochondrial morphologies have been identified and suggested to be important for particular tissue functionality and vitality (Scalettar et al., 1991; Hara et al., 2014; Glancy et al., 2015; Brandt et al., 2017; Figure 2), yet the underlying mechanisms and causalities are still unclear. For example, in the liver, individual mitochondrial structures are seen as compact, spherical shapes (Scalettar et al., 1991; Brandt et al., 2017). However, upon cellular stress, liver mitochondria change their spherical morphologies into either donut- or c-shaped formation (Ding et al., 2012), and aged liver tissues also show various empty spaces in the center of mitochondria (Brandt et al., 2017). In the brain, several investigations have revealed how mitochondrial morphology and function are related. For instance, mitochondria in the white matter of the mature mouse brain are elongated, tubular structures, but during the aging process, there is a reduction in mitochondrial number and an increase in length within these central tissues (Stahon et al., 2016). In this study, they also claimed that aging-related oxidative stress and impaired energy production may be linked with the alteration of mitochondrial morphologies in the brain tissues (Stahon et al., 2016). Meanwhile, it has been also suggested that mitochondrial morphology plays a key role for cognitive function. In the prefrontal cortex of monkey, both aging and deleted estrogen effects (i.e., ovariectomized) resulted in many donut-shaped mitochondrial formations at the presynaptic area where the size of active zones, as well as the number of docked vesicles, are smaller than in the brain of young or non-ovariectomized animals (Hara et al., 2014). Skeletal muscles have unique mitochondrial structures which are highly elongated and branched and contribute to the formation of a mitochondrial reticulum (Bubenzer, 1966; Bakeeva et al., 1978; Ogata and Yamasaki, 1985; Kirkwood et al., 1986; Glancy et al., 2015). It has been also reported that mitochondrial morphology is significantly related to muscle oxidative and calcium cycling capacities, as oxidative muscle fibers have larger volumes of individual mitochondria than glycolytic muscle fibers while glycolytic fibers have increased relative surface areas which allows for better exchange of calcium and other ions or metabolites with the cytosol (Bleck et al., 2018). Those observations suggest that mitochondrial structures are tightly linked with tissue quality and function.

## Mitochondrial Network Structure

**FIGURE 3:**
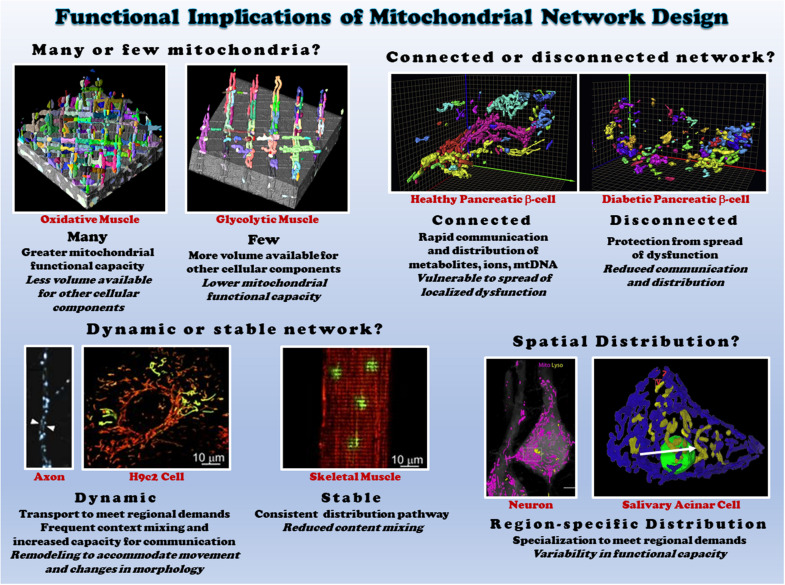
Functional consequences of different mitochondrial network structures. Top left: the size of the mitochondrial network determines the relative mitochondrial functional capacity within the cell. Shown are individual mitochondria (various colors) within oxidative (left) and glycolytic (right) muscle mitochondrial networks from (Bleck et al., 2018). Top right: Connectivity of mitochondrial networks enables rapid distribution. Mitochondrial networks from healthy (left) and diabetic (right) pancreatic β-cells are shown from (Ježek and Dlasková, 2019). Bottom left: mitochondrial networks can be dynamic or relatively stable. Mitochondria in axons (left from Misgeld et al., 2007) and H9c2 cells (middle from Eisner et al., 2014) are highly dynamic, while skeletal muscle mitochondria (right from Eisner et al., 2014) are relatively stable. Bottom right: regional variations in mitochondrial distribution allows for subcellular specification of mitochondrial function. Neurons (left from Gao et al., 2019) and salivary acinar cells (right from Porat-Shliom et al., 2019) both display regional heterogeneity of mitochondrial networks. All images reproduced with permission.

In cells, mitochondria can exist as either punctate, individual mitochondria or branched, highly connected architectures (Bleck et al., 2018; Valente et al., 2019) termed the mitochondrial network or reticulum (Figure 3). For example, mitochondria in vascular smooth muscles are ovoid or rod-shaped (Nixon et al., 1994; McCarron et al., 2013). On the other hand, the endothelium contains a tubular mitochondrial network (Shenouda et al., 2011). The importance of mitochondrial networks and connectivity in energy generation and signaling, as well as the significance of crosstalk between mitochondrial networks and other organelles, is being increasingly recognized. Mitochondrial network disruption causes mitochondrial dysfunction and has been implicated in multiple disorders (Annesley and Fisher, 2019), though some cell types, such as the smooth muscles mentioned above, normally operate in the absence of a mitochondrial network. Mitochondrial networks are complex, cell-type specific configurations that vary depending on cellular function and energy needs.

The mitochondrial reticulum enables flow of ions, proteins, metabolites, and mtDNA as well as efficient conduction of energy. The relatively stable, highly connected nature of mitochondria in both cardiac and skeletal muscles near the sarcolemma and between the myofibrils have been shown to support distribution of the electrical energy of the mitochondrial membrane potential rapidly throughout the cell (Amchenkova et al., 1988; Glancy et al., 2015, 2017; Bleck et al., 2018). However, just as cristae and individual mitochondria can be highly dynamic as discussed above, the electrical connectivity of mitochondrial networks also appears capable of dynamic responses to changing cellular environments. Localized dysfunction within mitochondrial networks induced by spatially controlled mitochondrial depolarization was found to result in rapid (5-10 s) disconnection of damaged mitochondria from the remainder of the network in striated muscle cells (Glancy et al., 2017). Seemingly, in contrast to these results, hypoxia and nutrient stress were found to lead to heterogeneous mitochondrial membrane potentials throughout the cardiac cell and localized ROS bursts led to synchronized, cell-wide oscillations in membrane potential and other energetic intermediates (Romashko et al., 1998; Aon et al., 2003, 2004; Juhaszova et al., 2004). However, these oscillations occurred ∼45 s after stress which is much slower than the network disconnection kinetics demonstrated for cardiac cells above. Further, more recent work by the same group (Kurz et al., 2010) showed that these spatio-temporal oscillations of membrane potential occurred in synchronized clusters of many neighboring mitochondria, leading to the conclusion that local coupling between neighboring mitochondria is dynamic in nature.

Furthermore, in response to external stimuli, cells can also alter their functional state via changes in mitochondrial network configuration. In yeast, there is an increase in mitochondrial networks and mitochondrial protein contents when yeast cells are in transition from a non-respiratory to respiratory state (Ohlmeier et al., 2004; Rafelski, 2013). Similarly, live cell imaging in HeLa cells revealed that change in energy substrates from glycolytic to oxidative is accompanied by both tubule branching and thinning in the mitochondrial networks (Rossignol et al., 2004).

### Functional Significance of Mitochondrial Network Structure

Mitochondrial network arrangement and the position of each mitochondrion in various cell types and tissues depend on the physiology and energy demand of the cell. In some cell types, mitochondria behave as dynamic networks, frequently changing network shape and subcellular distribution (Amchenkova et al., 1988; Bereiter-Hahn, 1990; Yaffe, 1999; Skulachev, 2001; Westermann and Prokisch, 2002). In cardiac and skeletal muscles of vertebrates, mitochondria are arranged into a highly organized, relatively stable mitochondrial reticulum (Bakeeva et al., 1978; Ogata and Yamasaki, 1985; Kirkwood et al., 1986; Weibel and Kayar, 1988; Glancy et al., 2015; Bleck et al., 2018). Likely in pancreatic cells and HL-1 cells with a cardiac phenotype, yeast cells show a dynamic mitochondria reticulum of branched tubules surrounding the nucleus (Hermann and Shaw, 1998; Egner et al., 2002; Karbowski and Youle, 2003). In neurons, where there is high energetic demands, there is a constant movement of mitochondria from cell body to synaptic sites with heterogeneous mitochondrial networks (Ligon and Steward, 2000; Miller and Sheetz, 2004; Miller et al., 2006). On the other hand, mitochondria in hepatocytes are more uniformly distributed throughout the whole cell (Brandt et al., 1974). *In vitro* culture of HL-1 cells with differentiated cardiac phenotype reveals a regular mitochondrial arrangement dissimilar to the native cardiac tissue mitochondrial structure, wherein dynamic dense mitochondrial reticulum undergo continual displacement and reorganization (short spheres or long filamentous mitochondria) (Kuznetsov et al., 2006; Pelloux et al., 2006). These pieces of evidence strongly suggest the functional and physiological relevance of mitochondrial network organization in cells and tissues.

Mitochondrial network morphology is not only specific to cell types but also demonstrates intracellular heterogeneity. Within a cell, mitochondria positioned at different site-specific regions display different morphology and biochemical properties (Romashko et al., 1998; Kuznetsov et al., 1998, 2004a, 2006; Collins et al., 2002; Collins and Bootman, 2003; Glancy et al., 2015; Benador et al., 2018; Porat-Shliom et al., 2019; Willingham et al., 2019). For example, mitochondrial populations close to the plasma membrane play an important role in functional coupling of ATP guided ion channels (e.g., Ca^2+^ entry) (Lawrie et al., 1996). Perinuclear mitochondrial networks regulate gene transcription by increasing ROS in the nucleus under hypoxia in intact lungs and cultured pulmonary artery endothelial cells (Al-Mehdi et al., 2012). In pancreatic acinar cells, mitochondria are organized into three functionally distinct groups located at the peripheral basolateral region near the plasma membrane, surrounding the nucleus, and between the granular area and basolateral region (Park et al., 2001). Specific subsets of mitochondrial networks and mitochondrial populations within cell types display dissimilar responses to substrates and inhibitors, as well as varied resistance or sensitive to oxidative stress, apoptosis or pathology (Romashko et al., 1998; Kuznetsov et al., 2004b; Chen et al., 2005; Willingham et al., 2019). A wide spectrum of cells (hepatocytes, HUVEC, astrocytes, HL-1 cells, fibroblasts and cultured human carcinoma cells) and tissues (cardiac, skeletal muscles and liver) display heterogeneous mitochondrial networks (Collins et al., 2002; Kuznetsov et al., 2004a, 2006; Bleck et al., 2018; Glancy, 2020). These findings highlight the extent of mitochondrial network complexity and diversity, which is likely involved in the diverse responses to various environmental stress and pathological processes across cell types.

### Mitochondrial Network Remodeling and Maintenance

The remodeling of dynamic mitochondrial networks depending on the changing physiological states of the cells or tissues is achieved by mitochondrial biogenesis, transport, and mitophagy, as well as by fusion and fission (Westermann, 2011; Labbe et al., 2014). Maintenance of mitochondrial network architecture via fusion and fission is important for proper mitochondrial and cellular function (Chen and Chan, 2005). *In vitro* experiments using digitonin-permeabilized cells fueled with substrates for ETC complexes I, II, and IV revealed that mitochondrial fragmentation caused by deficiency or absence of fusion factors like OPA1 and MFN1/2 results in significant reduction in mitochondrial respiratory capacity (Chen et al., 2005). On the other hand, yeast double mutants of genes responsible for fission and fusion showed interconnected mitochondrial networks as seen in wild-type cells (Sesaki and Jensen, 1999). Thus, although imbalance in fusion and fission can affect mitochondrial network configuration and function, fusion and fission factors are not solely responsible for mitochondrial network formation and maintenance. Several accessory proteins interact with core fusion and fission machinery to maintain mitochondrial network configurations (Cid-Castro et al., 2018; Yu and Pekkurnaz, 2018). Further, mitochondria are tethered to cytoskeletal elements and other organelles (Anesti and Scorrano, 2006; Schorr and van der Laan, 2018) (and discussed in detail below) and these interactions are crucial in determining mitochondrial network organization and function within the cell. Thus, the maintenance and functioning of mitochondrial networks not only involve the components of mitochondrial biogenesis, mitophagy, fusion, and fission, but are also regulated by various other factors. Further investigation is needed to identify the cellular factors that govern the formation and maintenance of mitochondrial networks.

### Mitochondrial Network Dysfunction

Characterizing the link between defects in mitochondrial network organization and mitochondrial dysfunction is particularly important in the context of aging and pathophysiological conditions. Recently, studies have explored the effect of mutations in mtDNA on mitochondrial network formation in human muscles and found that mitochondrial networks with mutant mtDNA form highly branched networks with increased nanotunnels between mitochondria (Vincent et al., 2019). In patients with mutations in respiratory-chain subunits, defective energy production was accompanied by mitochondrial network fragmentation in fibroblasts (Capaldi et al., 2004; Koopman et al., 2005). Interestingly, in fibroblasts, the inhibition of oxidative phosphorylation through respiratory chain complex I using rotenone perturbed mitochondrial network structure (Benard et al., 2007). Increased mutational heteroplasmy and mitochondrial stress also led to fragmentation in the mitochondrial network, whereas metabolic starvation induced increased fusion and mitochondrial tubulation (Gomes and Scorrano, 2011; Rambold et al., 2011).

Within mitochondrial networks, structural connectivity and energy conversion are interlinked; knockdown of fission factor, Drp1, in HeLa cells resulted in mitochondria that produced lesser energy (Benard et al., 2007). Mutations in genes responsible for mitochondrial fusion or fission affect mitochondrial network organization and also inhibit energy metabolism (Amati-Bonneau et al., 2005; Pich et al., 2005). In neurons, inhibition of mitochondrial fusion by OPA1 knock-out causes mitochondrial network fragmentation that is associated with mitophagy and defects in axonal transport (Twig et al., 2008a; Gomes and Scorrano, 2011). Ablation of mitochondrial fusion and fission leads to severe defects in mitochondrial function and metabolism in cardiac or skeletal muscles (Song et al., 2017). Studies in model organisms like Drosophila indicate that fragmented mitochondria resulting from loss of fusion factors are dysfunctional and negatively affect muscle physiology (Rai et al., 2014). Mitochondrial inflammatory myopathy caused by ablation of Opa1 in muscles is characterized by fragmented mitochondria (Rodriguez-Nuevo et al., 2018). In human skeletal muscles, mutation in fusion protein, Opa1, leads to abnormal mitochondrial morphology associated with reduced oxidative phosphorylation and ATP production (Lodi et al., 2004). However, as mentioned above, more work is needed to unravel whether the specific functional effects observed in mitochondrial dynamics knockdown models are due to alterations in the mitochondrial quality control system or to changes in mitochondrial structural configuration.

## Mitochondria-Organelle Interactions

**FIGURE 4:**
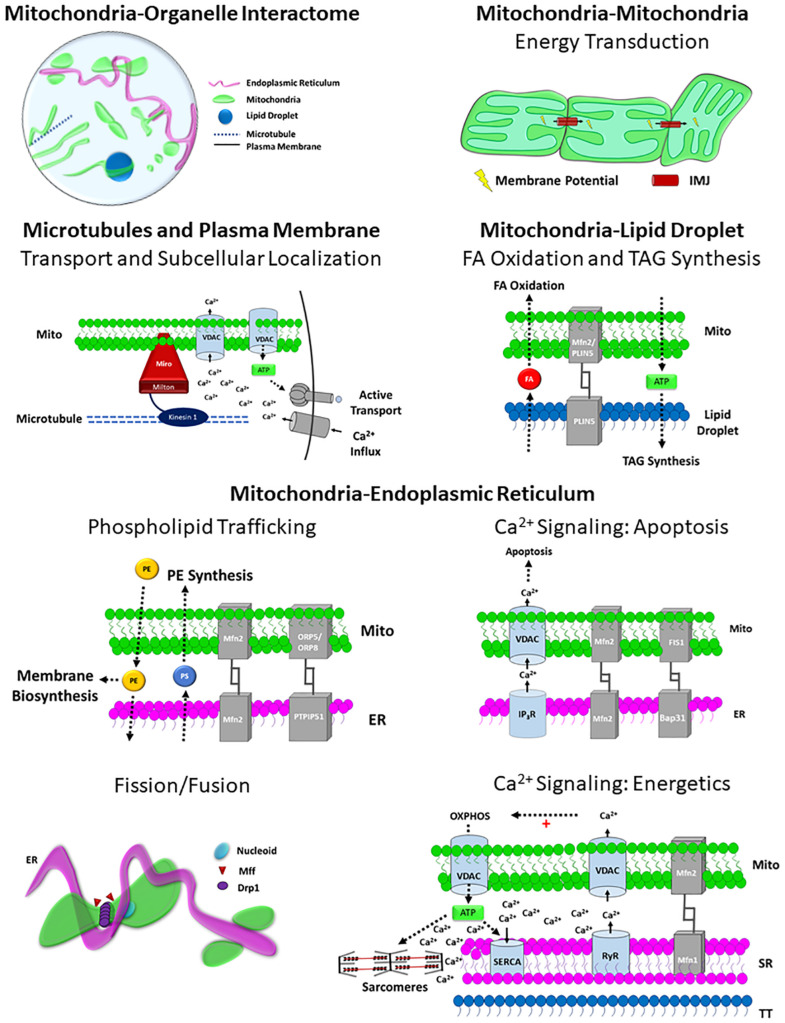
Structure-Function relationships of the Mitochondrial Interactome. Mitochondria-organelle interactions support cellular communication and facilitate many different processes related to energy metabolism, biosynthesis, and mitochondrial division through specialized structure-function relationships, and the topology of the overall mitochondrial-organelle can be specialized according to cell type and adaptable to changes in cellular energy homeostasis. The specific functions of mitochondria-organelle interactions are regulated by the transmembrane channels and tethering proteins that comprise the physical structure of the interorganelle contact site. Intermitochondrial junction (IMJ); Voltage-dependent anion channel (VDAC); Adenosine Triphosphate (ATP); Mitofusin 1 (MFN1); Mitofusin 2 (MFN2); Perilipin 5 (PLIN5); Phosphatidylserine (PS); Phosphatidylethanolamine (PE); Fatty acid (FA); Oxysterol-binding protein–related protein 5/8 (ORP5/8); Protein tyrosine phosphatase-interacting protein 51 (PTPIP51); Inositol-1,4,5-trisphosphate receptors (IP3R); β-cell receptor-associated protein 31 (Bap31); Mitochondrial fission 1 protein (Fis1); Dynamin related protein 1 (DRP1); Mitochondria fission factor (Mff); Ryanodine receptor (RyR); Sarco/endoplasmic reticulum Ca^2+^-ATPase (SERCA).

Because mitochondria serve critical roles in energy conversion, calcium cycling, lipid synthesis, and autophagy, it is imperative that mitochondria coordinate cellular processes with other organelles. While mitochondria-organelle contact sites have been observed for decades, recent advancements in cellular imaging techniques have revealed a complex organelle interactome in which mitochondria form functional contact sites with multiple organelles including the endoplasmic reticulum, lipid droplets, plasma membrane, microtubules, lysosomes, and endosomes. Mitochondria-organelle interactions facilitate cellular communication through the exchange of lipids, Ca^2+^, and other metabolites, and regulate mitochondrial metabolism, division, and biosynthesis through specialized (inter-organelle) structure-function relationships (Figure 4). The specific function of mitochondria-organelle interactions is regulated by the physical structure of the contact site, and studies across multiple models have found that the landscape of mitochondrial-organelle interactions is specialized according to cell type and adaptable to changes in cellular energy homeostasis. Moreover, mitochondria in contact with other organelles exhibit distinct protein expression profiles and functional capacities providing another mechanism by which structure-function relationships influence mitochondrial biology.

### Mitochondria-Endoplasmic Reticulum Contacts

The endoplasmic reticulum (ER) supports many cellular processes and closely works with the mitochondria to synchronize several biosynthesis and cell signaling pathways. Contact sites between mitochondria and the ER have been reported for decades across many cell types (Morre et al., 1971; Vance, 1990), and these interactions have been shown to largely support lipid synthesis and calcium transport. However, advances in live cell imaging strategies have revealed unique structure-function relationships of mitochondria-ER contact sites (Rizzuto et al., 1998; Friedman et al., 2011; Lewis et al., 2016; Valm et al., 2017; Guo et al., 2018), and recent studies have demonstrated how interactions between mitochondria and ER can regulate more complex cellular processes such as mitochondrial dynamics and DNA replication (Friedman et al., 2011; Lewis et al., 2016).

Mitochondria contact the ER more than any other organelle in the cell (Valm et al., 2017), and in cells with high calcium cycling demands, such as skeletal muscle, over 97% of the mitochondria are in contact with the ER (Bleck et al., 2018). Moreover, despite constant remodeling of the ER network (Nixon-Abell et al., 2016; Guo et al., 2018), studies in primate fibroblasts have demonstrated that contact sites between mitochondria are maintained during organelle trafficking and cellular stress (Friedman et al., 2010, 2011; Valm et al., 2017) and that the perseverance of mitochondria-ER contact sites during mitochondrial trafficking can itself serve as a mechanism by which ER remodeling occurs (Friedman et al., 2010; Guo et al., 2018). The tight connections formed between ER and mitochondria are maintained by tethering proteins such as protein tyrosine phosphatase-interacting protein 51 (PTPIP51) (De Vos et al., 2012), Mfn2 (de Brito and Scorrano, 2008), and Fis1 (Iwasawa et al., 2011) that are expressed on the outer membrane of mitochondria in contact with the ER. While mitochondria-ER interactions are abundant across many cell types, specific structure-function relationships at the site of contact can alter the biological function of these interactions in different cell types and conditions.

The proximity of the mitochondria and ER membranes allows for rapid, controlled exchange of ions between these organelles, and the role of mitochondria-ER interactions in cellular Ca^2+^ signaling has been historically appreciated (Giorgi et al., 2018). Structurally, Ca^2+^ exchange at the mitochondria-ER contact sites is enabled by voltage-dependent anion channels (VDACs) and mitochondrial-calcium uniporter (MCU) on the outer and inner membrane of the mitochondria, respectively, as well as the inositol-1,4,5-trisphosphate receptors (IP_3_R) and the sarcoplasmic reticulum ATPase (SERCA) expressed on the outer membrane of the ER (Szabadkai et al., 2006; Chami et al., 2008). Upon activation, IP_3_R releases Ca^2 +^ from the ER, creating a localized bolus of Ca^2+^ within the cytosol at the surface of the ER. While the affinity of VDAC to Ca^2+^ is relatively low, the tight tethering of mitochondria to the ER holds the membranes in close proximity and therefore mitochondrial VDACs are exposed to high regional Ca^2+^ concentrations during IP_3_R activation, which enables mitochondrial Ca^2+^ uptake (Rizzuto et al., 1993, 1998; Rapizzi et al., 2002). While IP_3_R activation is regulated by many cellular signaling pathways, the exchange of Ca^2+^ between the mitochondria and ER can be regulated by the physical characteristics of mitochondria-ER contact sites. Specifically, studies in cultured HeLa cells and myotubes have shown that increasing either VDAC expression or cytosolic-facing domain of IP_3_R in these cells is associated with a greater and more rapid increases in mitochondrial Ca^2+^ concentration immediately following stimulated ER Ca^2+^ release (Rizzuto et al., 1998). Moreover, studies evaluating the effect of tethering distance between mitochondria and ER in basophils found that mitochondrial Ca^2+^ could be enhanced by pulling the organelles closer together using synthetic linkers (Csordas et al., 2006), demonstrating how the spatial dynamics of mitochondria-ER contact sites can directly modulate interorganelle Ca^2+^ flux within these microdomains.

Calcium signaling is pivotal to cellular communication, and therefore the structure-function relationships of mitochondria-ER contact sites can serve as a regulatory mechanism for many cellular processes. For example, mitochondria can initiate apoptosis through the release/activation of cytochromes, caspases, DNase, and other apoptosis inducing factors (Hajnoczky et al., 1999), and experiments in mammalian cell lines have demonstrated how the structure and function of mitochondria-ER contact sites can regulate these pathways through Ca^2+^ exchange. Experiments in HeLa cells and basophils have demonstrated that exposure to apoptotic stimuli increases mitochondria-ER contact and decreases the distance between these organelles at the sites of interaction, resulting in increased Ca^2+^ uptake (Hajnoczky et al., 1999; Pinton et al., 2001; Csordas et al., 2006; Bravo et al., 2011). Apoptotic interactions between mitochondria and ER are stabilized and activated by FIS1-Bap31 tethering complex and IP_3_R-VDAC Ca^2+^ exchange (Iwasawa et al., 2011). Moreover, imaging studies in HeLa cells have found that increases in mitochondrial Ca^2+^ uptake following apoptotic stimuli can induce mitochondrial swelling and fragmenting of the mitochondrial network (Pinton et al., 2001). So, although mitochondrial-ER contact sites can provide Ca^2+^ buffer system under normal conditions, changes in these contact sites during periods of cell stress may cause large increases in mitochondrial Ca^2+^,which can lead to mitochondrial permeability and the induction of apoptotic pathways.

In striated muscle cells, mitochondria-ER Ca^2+^ exchange is also associated with the transient release of Ca^2+^ from the sarcoplasmic reticulum (SR) during muscle contraction, but in this system, the Ca^2+^ extrusion from the SR is regulated by the ryanodine receptors (RyR). The RyR is expressed on the outer membrane of the SR and is activated in either a Ca^2+^ (heart) or voltage (skeletal muscle) dependent manner following action potential propagation down the T-tubule (TT) system. Mitochondria-ER contact sties in striated muscle cells localize mitochondria to the dyad and triad SR-TT structures in heart and skeletal muscle, respectively, creating a mitochondria-SR-TT microdomain (Franzini-Armstrong et al., 1998, 2005). Therefore, similar to the IP3R-mediated Ca^2+^ release, mitochondrial VDAC is activated during muscle contraction by transient increases in Ca^2+^ concentrations within the mitochondria-SR-TT microdomains. Indeed, mitochondrial Ca^2+^ uptake has been shown to increase flux through multiple metabolic pathways, including oxidative phosphorylation, and the coupling of muscle contraction with mitochondrial Ca^2+^ striated muscles may provide a feedforward mechanism to support increases in cellular energy demand during muscle contraction (Murphy et al., 1990; Territo et al., 2000; Wust et al., 2011; Glancy et al., 2013). Furthermore, diastole and skeletal muscle relaxation require ATP-dependent Ca^2+^ reuptake into the SR, and the colocalization of mitochondria and SR provides rapid diffusion cellular free energy to SERCA at the surface of the SR (Territo et al., 2000; Franzini-Armstrong et al., 2005; Bleck et al., 2018).

Cellular lipid homeostasis is also supported by specialized phospholipid synthesis and trafficking mechanisms at the mitochondria-ER contact sites. Mitochondria cannot independently synthesize specific phospholipids such as phosphatidylserine (PS) and phosphatidic acid that serve as precursors for phosphatidylethanolamine (PE) and cardiolipin, respectively, and therefore rely on the ER-mediated biosynthesis of these lipids. While high resolution images have revealed that mitochondria-ER contact sites are not coupled to the ribosome secretory pathway (Csordas et al., 2006), mitochondria-ER contact sites provide efficient, non-vesicular lipid transport between these organelle (Vance, 1990; Vance and Tasseva, 2013). In yeast, studies have demonstrated the role of ER-mitochondria encounter site (ERMES) as the major player in mitochondria-lipid transport (Kornmann et al., 2009; Toulmay and Prinz, 2012; Schauder et al., 2014), but the ERMES complex are not evolutionarily conserved in mammalian cells. Studies in HeLa cells have found that the phospholipid exchanging proteins ORP5 and ORP8 localize to mitochondria-ER contact sites and associate with PTPIP5 (Chung et al., 2015; Galmes et al., 2016) and that ORP5/8 depletion results in alterations in mitochondrial ultrastructure including robust declines in cristae formation as well as reductions in energetic capacity (Galmes et al., 2016). Notwithstanding, these studies did not directly evaluate lipid trafficking, and the mechanisms of phospholipid transfer between the mitochondria and ER in mammalian cells remain unknown. In addition to supporting PE and cardiolipin biosynthesis, lipid trafficking between the mitochondria and ER also facilitates autophagosome formation (Hailey et al., 2010; Gomes et al., 2011; Rambold et al., 2011, 2015; Gomez-Suaga et al., 2017; Nguyen et al., 2017). Studies in mammalian cell lines have found that, during periods of starvation, autophagosomes form at mitochondria-ER contact sites in an Mfn2-dependant manner (Hailey et al., 2010). Further experiments using a fluorescently labeled PS analog determined that ER-derived PS was shuttled from the ER into the mitochondria where it was subsequently converted into PE and used to construct the membranes of newly formed autophagosomes (Hailey et al., 2010). Interestingly, autophagosomes are not associated with mitochondria-ER contact sites during periods of ER-stress and may even attenuate autophagy under basal conditions, suggesting that the role of mitochondria-ER lipid trafficking in the formation of autophagosomes is specific to autophagic pathways activated during periods of nutrient deprivation (Hailey et al., 2010; Stoica et al., 2014; Gomez-Suaga et al., 2017).

In addition to ion and lipid trafficking, mitochondria-ER interactions can facilitate mitochondrial dynamics and events and studies in yeast and mammalian fibroblasts have found that more 80% of mitochondrial fission events occur at mitochondria-ER contact sites (Friedman et al., 2011; Guo et al., 2018). Live cell experiments have revealed that the mitochondria-ER contact sites associated with mitochondrial division are defined by unique physical interactions between these organelle where the ER tubules cross over and constrict the mitochondria (Friedman et al., 2011; Guo et al., 2018). In yeast, ER tubules nearly circumscribe mitochondria and constricted the diameter of the mitochondria by more than 30% (Friedman et al., 2011). While the 3D spatial dynamics of mitochondria-ER division is less understood, time course imaging studies in mammalian cell lines have demonstrated that mitochondrial-ER contact sites are maintained throughout the division of mitochondria at the site of ER-mediated constriction. Furthermore, mitochondria-ER interactions associated with mitochondrial division are spatially coupled to nucleoids with actively replicating mtDNA in mammalian systems, suggesting that these contact sites coordinate divisions at the outer and inner mitochondrial membranes (Lewis et al., 2016). While mitochondrial division proteins Drp1 and Mff colocalize to mitochondria-ER interactions and are necessary for mitochondrial division, experiments in primate fibroblasts depleted of Drp1 and Mff found that these proteins are not required for ER-mediated mitochondrial constriction (Friedman et al., 2011; Lewis et al., 2016). Moreover, constriction of mitochondria by ER is also associated with mitochondrial fusion, and studies in mammalian cells have found that fusion events at mitochondria-ER contact sites occur more often as 43% faster compared to mitochondrial fusion events not associated with ER (Guo et al., 2018). Although the role of mitochondria-ER interactions in mitochondrial kinetics remains elusive, the preponderance of evidence suggests that constriction of mitochondria by ER facilitates fission and fusion events by decreasing the mitochondrial surface area at this interface and thereby enabling the actions of the division machinery and expediting the coalescence of mitochondrial membranes.

### Mitochondria-Lipid Droplet Contacts

Mitochondria metabolize, synthesize, and structurally incorporate lipids, and it is therefore not surprising that mitochondria-lipid droplet interactions are predominant across numerous cell types including fibroblasts, striated muscles, brown fat, and others. While mitochondria-lipid droplet interactions represent a significant portion of the organelle interactome, these interactions are dynamic and the frequency and function of mitochondria-lipid droplet interactions vary across cell types and metabolic states. Lipid droplets serve as cellular storage for fatty acids (FAs) in the form of triacylglycerides (TAGs), but the biological function of mitochondria-lipid interactions may support either metabolism or synthesis of TAGs depending on the cell type and metabolic status. For example, in cells with high energy demands, mitochondria-lipid interactions support cellular energy homeostasis by breaking TAGs down into FAs and importing them into the mitochondria to be used in FA-oxidation (Tarnopolsky et al., 2007; Shaw et al., 2008; Wang et al., 2011; Cabodevilla et al., 2013; Herms et al., 2015; Rambold et al., 2015; Bleck et al., 2018). Alternatively, other mitochondria-lipid interactions are designed to power ATP-dependent TAG synthesis with mitochondrial oxidative phosphorylation (Benador et al., 2018). Lipid droplets can also protect mitochondria from FA toxicity by buffering FAs from the cytosol and the interactions between these organelle can adapt to periods of metabolic stress (Herms et al., 2015; Rambold et al., 2015; Nguyen et al., 2017).

Depending on the function of mitochondria-lipid droplet interactions, the frequency and structure of these contact sites can be modulated to sustain energy homeostasis across different cell type and metabolic states. Studies evaluating lipid-organelle interactions in primate fibroblasts have found that 20% of the lipid droplets form contact sites with mitochondria and demonstrated that these interactions directly facilitate the transport of FAs from LDs into mitochondria for FA-oxidation (Valm et al., 2017). Moreover, functional experiments of FA metabolism in these fibroblasts demonstrated that the level of interaction between lipid droplets and mitochondria can increase ∼10% following nutrient deprivation to sustain the increased reliance on FA metabolism (Valm et al., 2017). Mitochondria-lipid droplet interactions also support FA metabolism in striated muscles cells, and high resolution electron microscopy images of murine striated muscles have shown that 20-50% of the mitochondria are in contact with lipid droplets and that the distribution of these interactions across muscle cell types is directly related to energetic capacity (Bleck et al., 2018). Specifically, mitochondria-lipid droplet interactions are more than twice as prevalent in cardiomyocytes compared to cells from slow-twitch oxidative skeletal muscle cells; whereas essentially no mitochondria-lipid interactions are found in cells from fast-twitch glycolytic muscles (Bleck et al., 2018). Similarly, studies evaluating lipid droplet interactions in human skeletal muscle cells have found that 50-75% of the lipid droplets interact with mitochondria and reported a 12-21% increase in mitochondria-lipid droplet interactions following exercise (Tarnopolsky et al., 2007). In other cell types, mitochondria-lipid droplet interactions support lipid synthesis, and the transmission of stored energy between these organelles is reversed. In tissues such as brown adipose tissues (BAT), 75% of the mitochondria are in contact with lipid droplets, but functional experiments in murine BAT cells demonstrated these contact sites support ATP-mediated TAG biosynthesis (Benador et al., 2018). Furthermore, increasing FA oxidation with cold exposure reduced the proportion of mitochondria touching lipid droplets by more than half and increases in mitochondria-lipid droplet interactions were associated with robust increases in lipid droplet size (Benador et al., 2018).

While altering the frequency of contact sites between mitochondria and lipids provides a mechanism for throttling cellular FA flux, the precise functions of mitochondria-lipid interactions are determined by the protein expression and morphology of the mitochondria participating in these points of contact. Although less is known about mitochondrial tethering mechanisms associated with lipids compared to the ER, it has been shown that mitochondria express tethering proteins such as Plin5 and Mfn2 on the OMM at lipid sites (Granneman et al., 2011; Wang et al., 2011; Bosma et al., 2012, 2013; Benador et al., 2018), and increases in the expression of these tethers are associated with increased mitochondria-lipid interactions. In addition, mitochondria-lipid contact sites that support the import of FA into the mitochondria are comprised of specific machinery required for TAG metabolism such as adipose triglyceride lipase (ATGL) (Smirnova et al., 2006; Rambold et al., 2015), and some studies suggest that simultaneous TAG synthesis and ATGL-mediated lipolysis can regulate FA flux and therefore protect mitochondria from FA toxicity during starvation and periods of autophagy (Nguyen et al., 2017).

Mitochondria in contact with lipid droplets also exhibit distinct morphology that supports the function of the interaction. In mature muscle cells, large, elongated mitochondria wrap around the lipid droplets to increase surface area of contact between the organelle (Tarnopolsky et al., 2007; Shaw et al., 2008; Wang et al., 2011; Bleck et al., 2018) and are highly connected to surrounding mitochondria. Similarly, studies in MEFs have found that increases in FA metabolism are associated with the formation of fused, tubular networks of mitochondria (Gomes et al., 2011; Rambold et al., 2011, 2015) and these connected networks directly enable for the distribution of LD-derived FAs throughout the mitochondria during periods of increased reliance on beta-oxidation. In BAT, mitochondria in contact with lipid droplets are also significantly larger and elongated, but in keeping with the TAG synthesis role of mitochondria-lipid droplet contacts in this tissue, functional studies found that mitochondria attached to lipid droplets actually have lower capacity to metabolize FAs and higher ATP production rates (Benador et al., 2018). Together, these experiments demonstrate how the regularity, structure, and function of mitochondria-lipid interactions can regulate cell-type specificity and subcellular specialization of mitochondrial function.

### Mitochondria-Microtubule and Plasma Membrane Contacts

Mitochondrial motility and spatial distribution within cells can be enabled, in part, through interactions with the microtubules (MT) and plasma membranes (PM). The capacity to transport and spatially distribute mitochondria within the cell allows for localized regulation of metabolism and calcium buffering as well as proper distribution of mitochondria during cell division. Long-distance transport of mitochondria is largely supported by mitochondria-MT interactions where anterograde and retrograde movement of mitochondria along microtubules is powered by kinesin and dynein motors (Morris and Hollenbeck, 1995; Pilling et al., 2006). In higher eukaryotes, mitochondria-MT interactions are comprised of a motor/adaptor complex in which the mitochondrial outer membrane protein Miro interacts with the kinesin motor Kinesin-1 through the adaptor protein Milton (Stowers et al., 2002; Guo et al., 2005; Glater et al., 2006; Wang and Schwarz, 2009). Long-distance mitochondrial transport is particularly critical in large, asymmetric cells with highly specialized subregions. In neurons, mitochondria are transported down axons along MTs to provide cellular free energy and calcium buffering support at sites of synaptic transmission and neurite growth (Morris and Hollenbeck, 1993, 1995; Chada and Hollenbeck, 2003, 2004), resulting in greater energy demands in neurite mitochondria compared to the cell body (Willingham et al., 2019). Indeed, the importance of mitochondrial-MT interactions in neural biology and pathology has been widely recognized (Saxton and Hollenbeck, 2012), and studies have demonstrated that altering mitochondria-MT interactions through deletion of the Miro1 adaptor protein in mice arrests mitochondrial motility and results in severe neurological disease (Nguyen et al., 2014). Mitochondria-MT interactions have also been shown to regulate cytosolic Ca^2+^ concentrations, and functional studies across multiple mammalian cell lines have found that mitochondrial movement along MTs is inhibited by increases in Ca^2+^ concentrations (Quintana et al., 2006, 2007; Wang and Schwarz, 2009), providing a mechanism to target mitochondria to cellular subregions with high cytosolic Ca^2+^ concentrations. Similarly, studies in T cells have demonstrated that mitochondria-MT interactions act to mobilize mitochondria to the plasma membrane at sites of high Ca^2+^ influx and support the function of critical ion channels by buffering excess Ca^2+^ within the microdomain of the immunological synapse (Quintana et al., 2006, 2007). Imaging studies in multiple different mammalian cells lines have also found that mitochondria-MT interactions also facilitate the localization of mitochondria to the plasma membrane prior to and throughout cell division (Hu et al., 2008; Lawrence and Mandato, 2013).

While mitochondria-MT interactions are often in close proximity with PM (Quintana et al., 2006, 2007; Hu et al., 2008; Lawrence and Mandato, 2013), the physical mechanisms underlying mitochondria-PM interactions in mammalian cells remain largely unknown. Indeed, mitochondria-PM interactions have been observed in non-dividing mammalian cells, suggesting that mitochondria-PM interactions have important functional implications that extend beyond mitochondria inheritance in dividing cells. For example, *in vivo* imaging studies of mouse salivary glands demonstrated that a subpopulation of highly fused mitochondria localize to the basolateral plasma membrane, indicating that mitochondria-PM interactions may also support subcellular distribution and functional specialization of mitochondria in non-dividing mammalian cells as well (Porat-Shliom et al., 2019). Although further experiments found that over half of the mitochondria in salivary glands were less than 2 μM from the plasma membrane and exhibited high spatiotemporal stability (Porat-Shliom et al., 2019), a precise mechanism of mitochondria-PM contact has been not identified. Mitochondria-PM interactions have been extensively characterized in yeast, and studies have revealed a specialized anchoring mechanism at mitochondria-PM contact sites that enable the proportionate distribution of mitochondria in dividing yeast cells. During cell division in yeast, mitochondria become fused, and the elongated mitochondria localize to the periphery of the cell where they are tightly fastened to the PM in conjunction with the ER and form the mitochondria-ER-cortex anchor (MECA) complex (Lackner et al., 2013). As with other mitochondria-organelle interactions, the MECA is supported by specialized tethers, and live cell imaging studies have revealed that the tethering proteins Num1 and Mdm36 are critical to the formation of the MECA and the distribution of mitochondria during cell division (Klecker et al., 2013; Lackner et al., 2013). Nonetheless, MECA is not conserved in higher eukaryotes, and the specific tethering systems responsible for linking mitochondria to the PM in differentiated cell types has not yet been characterized in mammals, although a recent study evaluating mitochondria-PM interactions in mammalian stem cells reported a potential tethering role for MFN1 (Wu et al., 2019).

### Mitochondria-Lysosome/Endosome Interactions

Lysosomes and endosomes interact with mitochondria to carry out cellular processes related to biosynthesis, intracellular trafficking, and degradation (Zhao et al., 2012; Daniele et al., 2014; Wong et al., 2018, 2019; Yamano et al., 2018; Cioni et al., 2019; Munoz-Braceras et al., 2019). Interactions between these organelles have been observed in many cells types from yeast to cardiomyocytes (Zhao et al., 2012; Daniele et al., 2014; Elbaz-Alon et al., 2014; Honscher et al., 2014; Wong et al., 2018, 2019; Yamano et al., 2018; Cioni et al., 2019; Munoz-Braceras et al., 2019), and studies have found that 15-30% of the lysosomes in mammalian cells are in contact with mitochondria (Valm et al., 2017; Wong et al., 2018). Lysosomes are primarily tethered to the mitochondria by the lysosomal membrane protein Rab7 (Zhang et al., 2005; Zhao et al., 2012; Wong et al., 2018; Yamano et al., 2018; Cioni et al., 2019; Munoz-Braceras et al., 2019; Wong et al., 2019). Depending on the nature of the interaction, mitochondria-lysosome interactions are supported by different proteins on the outer mitochondrial membrane such as MFN2 (Zhao et al., 2012; Daniele et al., 2014), the vacuolar protein sorting 13 (Vps13) (John Peter et al., 2017; Munoz-Braceras et al., 2019), or FIS1. Because lysosomal degradation pathways are critical to removing cellular debris, mitochondria-lysosome interactions have been largely studied in the context of autophagic pathways that remove damaged and/or malfunctional mitochondria (Youle and Narendra, 2011; Yamano et al., 2014, 2018; Pickrell and Youle, 2015; Das et al., 2016; Fermie et al., 2018). For example, Rab7-MFN2-mediated interactions between mitochondria and lysosomes facilitate the formation of autophagosome-lysosomes, and experiments in cultured cardiomyocytes have demonstrated increased Rab7-MFN2 interactivity during starvation-induced autophagy (Zhao et al., 2012). In addition, studies in mammalian cell lines have also shown that Vps13 colocalized to mitochondria-lysosome contact sites and that Rab7-Vps13 interactions modulate autophagy by facilitating the lysosomal degradation (Munoz-Braceras et al., 2015, 2019). As highlighted below, emerging evidence has demonstrated that mitochondria also form functional, non-autophagic interactions with lysosomes and endosomes in healthy cells (Han et al., 2017; Chen et al., 2018; Fermie et al., 2018; Wong et al., 2018, 2019).

Rab7 is a GTP-bound protein, and therefore, mitochondrial-lysosome interactivity can be modulated by GTPase activity at the mitochondria-lysosome contact site. The outer mitochondrial membrane protein Fis1 recruits the GTPase TBC1D15 to the organelle contact site which subsequently untethers the organelles via GTP hydrolysis (Zhang et al., 2005; Onoue et al., 2013; Chen et al., 2018; Wong et al., 2018; Yamano et al., 2018). Recent live cell imaging studies in HeLa cells have demonstrated that lysosomes interact with mitochondria via ∼80% of all mitochondrial fission events and that Fis1/TBC1D15-mediated Rab7 GTPase activity functions to modulate mitochondria-lysosome interactions during fission (Wong et al., 2018). Specifically, inhibition of TBC1D15-mediated Rab7 GTPase activity significantly reduces mitochondrial fission and results in elongated, overly fused mitochondrial networks (Wong et al., 2018). These experiments also found that both ER and lysosomes interact with mitochondria at sites of fission, indicating multi-organelle coordination of membrane fission. In addition, studies in other mammalian cell lines have reported reduced glucose metabolism in Rab7 knockdown and TBC1D15 knockout cells (Ding et al., 2017; Wu et al., 2019), but the metabolic function of TBC1D15-mediated Rab7 GTPase activity remains unclear and further investigation is warranted.

Mitochondria may also interact with endosomes and lysosomes to facilitate interorganelle ion transfer (Sheftel et al., 2007; Das et al., 2016; Hamdi et al., 2016) and localized protein synthesis (Cioni et al., 2019). Similar to lysosomes, endosomes dynamically interact with mitochondria in healthy cells, and functional studies in red blood cells suggest that mitochondria-endosome interactions may enable iron transfer from iron-rich endosomes to mitochondria (Sheftel et al., 2007; Das et al., 2016; Hamdi et al., 2016). Indeed, the direct transfer of iron from endosomes to mitochondria could function to support mitochondrial iron-sulfur cluster biosynthesis and energy metabolism (Braymer and Lill, 2017). However, the precise components of the mitochondria-endosome contact site are unclear and the mechanism by which mitochondria-endosome iron exchange occurs has not been established. Interorganelle ion exchange has also been postulated as a function of mitochondria-lysosome interactions. Like mitochondria, lysosomes can act as intracellular Ca^2+^ storage centers, and studies in cultured mammalian cell lines have demonstrated that activation of the lysosomal membrane channel TRPML1 causes release of lysosomal Ca^2+^ (Zhang et al., 2016). Interestingly, TRPML1-activation was found to be induced by increases in cytosolic ROS (Zhang et al., 2016), thereby providing a direct link between lysosomal Ca^2+^ release and mitochondria function (Brand, 2016). Furthermore, applications of high-resolution 3D electron microscopy have also identified mitochondria-SR-lysosome microdomains in cardiomyocytes (Aston et al., 2017), suggesting that mitochondria-lysosome interactions may facilitate Ca^2+^ buffering/signaling pathways in striated muscle. However, the function of mitochondria-SR-lysosome interactions in muscle cells remains unclear. In addition, interactions between mitochondria and late-endosomes/lysosomes can also support subcellular localization of protein synthesis. For example, Rab7a-mediated interactions between mitochondria and late-endosomes have been shown to coordinate the translation and placement of mitochondrial membrane proteins at specific subcellular locations (Cioni et al., 2019). These experiments, performed in retinal ganglion cells, demonstrated that nuclear encoded mRNA is transported down axons on late-endosomes that localize to mitochondria where protein synthesis ultimately occurs (Cioni et al., 2019). Taken together, the current data indicate that mitochondria interact with lysosomes and endosomes to support a multitude of processes in healthy cells that extend far beyond the lysosomal degradation pathways associated with autophagy.

### Mitochondria-Mitochondria Contact Sites

Mitochondria can also form specific contacts with other adjacent mitochondria via intermitochondrial junctions (IMJs) that are featured by high electron density in electron microscopy images (Bakeeva et al., 1983; Davidowitz et al., 1984; Duvert et al., 1985; Picard et al., 2015; Glancy et al., 2017), though specific IMJ tethering proteins have not yet been elucidated. At IMJs, mitochondrial cristae orientation is often well aligned with the cristae of neighboring mitochondria, and it has been suggested that this trans-mitochondrial cristae coordination may allow for more efficient content transfer and communication (Picard et al., 2015). Formation of IMJs appears to be related to cellular energy demand as tissues facing high energy demands, such as heart and skeletal muscle, have more IMJs than other less demanding tissues (Picard et al., 2015). Additionally, it was shown that mitochondria coupled with IMJs are more elongated and larger compared to non-connected mitochondria in mouse heart cells where IMJs often link many mitochondria together along the longitudinal axis of the cardiomyocyte (Glancy et al., 2017). It was also noted that IMJs may play an important role for both cellular energy distribution and protection from spreading dysfunction throughout the mitochondrial network (Glancy et al., 2017). Moreover, outer mitochondrial membrane protein mitoNEET was recently shown to be critical for IMJ formation as well as mitochondrial respiratory function (Vernay et al., 2017). Nevertheless, more studies are warranted to better understand underlying mechanisms and functional implications of IMJ formation.

### Topology of the Mitochondria-Organelle Interactome

The diversity of functions exhibited across the various mitochondria-organelle contact sites demonstrates the complex role of subcellular architecture in cell-type specialization. Although the importance of structure-function relationships within the mitochondrial-organelle interactome is just beginning to be uncovered, it is clear that mitochondria-organelle interactions are a key determinant of cell function. Because of the finite surface area of mitochondrial outer membrane within the given cell, establishing contact sites physically reduces the potential for interactions with other organelles as well as with the cytosol, which may limit other functionalities. Thus, the topology of the mitochondria-organelle interactome must be optimized to accommodate the specific functions of a cell. This trade-off is well represented in the interactivity of mitochondria in striated muscles where interactions with lipid droplets and SR are evidently tuned to meet the Ca^2+^ cycling capacities of various muscle types (Bleck et al., 2018). However, the preponderance of evidence related to mitochondria-organelle interactions is derived from yeast and basic cell biology models that are limited in their ability to provide information related to physiological cell-type specialization of the mitochondrial interactome. Undoubtedly, studies evaluating cellular responses to metabolic and autophagic stressors have demonstrated the robust plasticity of the mitochondria-organelle interactome and revealed potential mechanisms by which cells might alter mitochondria-organelle interactions to achieve tissue-specific functionalities, but the extent to which mitochondrial interactivity is customized according to cell type remains unknown. Future studies evaluating the physiological regulation of the mitochondria-organelle interactome may provide new insights into cell-type specialization of mitochondrial function and novel mechanism of pathological mitochondrial dysfunction.

## Multiscale Coordination of Mitochondrial Structure and Function

As discussed above, physiological modulation of mitochondrial structure in support of the functional specificity of a cell can occur at several different spatial scales. While there have been many studies and subsequent reviews focusing on mitochondrial structure at each individual level, there have been relatively fewer which investigate how mitochondrial structure may be coordinated across scales within a cell (Lewis et al., 2016; Yang and Svitkina, 2019; Qin et al., 2020) and even less looking at how this coordination is modulated within physiological systems (Sood et al., 2014; Bleck et al., 2018; Ježek and Dlasková, 2019). In healthy pancreatic β-cells, dynamic networks are formed of many elongated, tubular mitochondria containing relatively flat, lamellar cristae and nucleoids distributed along their length (Ježek and Dlasková, 2019). However, in diabetic β-cell models, mitochondria become fragmented, form spherical shapes, and disconnect from the network (Dlasková et al., 2010). In addition, there is a loss of cristae and nucleoids begin to form clusters (Anello et al., 2005; Ježek and Dlasková, 2019). Moreover, diabetic β-cells from human donors were found to have fewer interactions between mitochondria (VDAC1) and ER (IP3R2) (Thivolet et al., 2017). Each of the above mitochondrial structure modifications in the diabetic β-cell suggests a loss in intracellular communication and signaling capacity (Figures 1-3) and highlights the consistency of the functional impact of the multi-scale mitochondrial structural response under these pathological conditions.

Multi-scale coordination of mitochondrial structure-function relationships can also occur on an acute timescale. Using a postprandial model in mice, mitochondrial morphology across spatial scales was compared in hepatocytes 2 and 5 h after feeding in order to relate energetic and biosynthetic function under conditions of varying anabolic/catabolic states in the liver (Sood et al., 2014). As mTORC1 activity fell during the later postprandial stage, there were a greater number of mitochondria which were smaller in size and more compact in shape though overall mitochondrial volume did not appear to change consistent with a fragmentation of the mitochondrial network rather than an acute change in the rate of mitochondrial biogenesis or mitophagy. Also similar to the β-cell diabetic response above, there was a loss of cristae density and cristae number in the later postprandial state with a concomitant loss in energy conversion capacity. However, unlike the diabetic β-cell, hepatocytes in the late postprandial state increased interactions between mitochondria and ER by more than doubling the length of mitochondria-ER contact sites along the perimeter of the mitochondrion. While this increase in interactions between mitochondria and ER was dependent on MFN2, the lack of information on the other tethering proteins involved makes it difficult to interpret the specific functional implications of the increased contact sites (Figure 4). It is tempting, however, to speculate that the increased mitochondria-ER contact sites may have facilitated the demonstrated fragmentation of the mitochondrial network (Sood et al., 2014). Either way, it appears that physiological remodeling of mitochondrial structures can be coordinated across several spatial scales to support functional specificity within the cell as either an acute or chronic adaptation.

## Conclusion

Mitochondria are remarkable organelles capable of remodeling their structural organization, sometimes in a nearly continuous fashion, in order to optimize mitochondrial function in support of cellular demands. As discussed above, the structural configuration of mitochondria can be regulated at several different levels to specifically tune mitochondria to the needs of the cell in which they reside. In a physiological setting, altering mitochondrial structure is associated with various functional trade-offs at each spatial scale. Indeed, the morphology within the interior of mitochondria, particularly the relative proportion and shape of mitochondrial cristae, determines the space available for enzymes involved in different functional pathways. While the overall volume of an individual mitochondrion is reflective of its internal functional capacity, the shape of a mitochondrion plays a large role in its ability to interact with the surrounding cellular environment. Moreover, cells can form mitochondrial networks with varying sizes, shapes, and dynamics each of which contribute to the overall functional capacity of mitochondria within the cell. Additionally, interactions between mitochondria and other organelles are modulated by distinct tethering proteins which regulate the specific functional coupling that occurs at these organelle contact sites. Finally, emerging evidence suggests that mitochondrial functional specificity of cells within tissues occurs through coordinated, multi-scale regulation of mitochondrial structure.

## Author Contributions

All authors drafted and edited the manuscript.

## Conflict of Interest

The authors declare that the research was conducted in the absence of any commercial or financial relationships that could be construed as a potential conflict of interest.

## Abbreviations

ATGL: adipose triglyceride lipase
ATP: adenosine triphosphate
BAP31: β-cell receptor associate protein 31
BAT: brown adipose tissue
Ca^2+^: calcium
Drp1: dynamin related protein 1
ER: endoplasmic reticulum
ERMES: endoplasmic reticulum-mitochondrial encounter site
ETC: electron transport chain
FA: fatty acid
Fis1: mitochondrial fission 1 protein
GTP: guanosine triphosphate
HUVEC: human umbilical vein endothelial cells
IMJ: intermitochondrial junction
IMM: inner mitochondrial membrane
IP_3_R: inositol-1,4,5-triphosphate receptors
LD: lipid droplet
MCU: mitochondrial calcium uniporter
MECA: mitochondria-endoplasmic reticulum-cortex anchor complex
MEF: mouse embryonic fibroblast
Mff: mitochondrial fission factor
Mfn1/2: mitofusin 1/2
MICOS: mitochondrial contact site and cristae organizing system
Mid49/51: mitochondrial dynamics proteins of 49/51 kDa
Miro: mitochondrial rho gtpase protein
MT: microtubule
mtDNA: mitochondrial deoxyribonucleic acid
mtPA-GFP: mitochondrial photoactivatable green fluorescent protein
OMM: outer mitochondrial membrane
Opa1: optic atrophy protein 1
ORP5/8: oxysterol binding protein related protein 5/8
PE: phosphatidylethanolamine
PLIN: perilipin
PM: plasma membrane
PS: phosphatidylserine
PTPIP51: protein tyrosine phosphatase interacting protein 51
ROS: reactive oxygen species
RyR: ryanodine receptor
SERCA: sarco/endoplasmic reticulum calcium atpase
SR: sarcoplasmic reticulum
TAG: triacylglycerol
TCA: tricarboxylic acid cycle
TBC1D15: TBC1 domain family member 15
TRPML1: transient receptor potential cation channel, mucolipin subfamily 1
TT: transverse tubule
VDAC: voltage dependent anion channel
VPS13: vacuolar protein sorting 13.

## References

[B1] Ahmad T. Aggarwal K. Pattnaik B. Mukherjee S. Sethi T. Tiwari B. K. (2013). Computational classification of mitochondrial shapes reflects stress and redox state. *Cell Death Dis.* 4:e461. 10.1038/cddis.2012.213 23328668 PMC3564000

[B2] Al-Mehdi A. B. Pastukh V. M. Swiger B. M. Reed D. J. Patel M. R. Bardwell G. C. (2012). Perinuclear mitochondrial clustering creates an oxidant-rich nuclear domain required for hypoxia-induced transcription. *Sci. Signal* 5:ra47. 10.1126/scisignal.2002712 22763339 PMC3565837

[B3] Amati-Bonneau P. Guichet A. Olichon A. Chevrollier A. Viala F. Miot S. (2005). OPA1 R445H mutation in optic atrophy associated with sensorineural deafness. *Ann. Neurol.* 58 958–963. 10.1002/ana.20681 16240368

[B4] Amchenkova A. A. Bakeeva L. E. Chentsov Y. S. Skulachev V. P. Zorov D. B. (1988). Coupling membranes as energy-transmitting cables. I. Filamentous mitochondria in fibroblasts and mitochondrial clusters in cardiomyocytes. *J. Cell Biol.* 107 481–495. 10.1083/jcb.107.2.481 3417757 PMC2115217

[B5] Anderson G. R. Wardell S. E. Cakir M. Yip C. Ahn Y. R. Ali M. (2018). Dysregulation of mitochondrial dynamics proteins are a targetable feature of human tumors. *Nat. Commun.* 9:1677. 10.1038/s41467-018-04033-x PMC5919970 29700304

[B6] Anello M. Lupi R. Spampinato D. Piro S. Masini M. Boggi U. (2005). Functional and morphological alterations of mitochondria in pancreatic beta cells from type 2 diabetic patients. *Diabetologia* 48 282–289. 10.1007/s00125-004-1627-9 15654602

[B7] Anesti V. Scorrano L. (2006). The relationship between mitochondrial shape and function and the cytoskeleton. *Biochim. Biophys. Acta* 1757 692–699. 10.1016/j.bbabio.2006.04.013 16729962

[B8] Annesley S. J. Fisher P. R. (2019). Mitochondria in health and disease. *Cells* 8 25–29. 10.3390/cells8070680 PMC6678092 31284394

[B9] Aon M. A. Cortassa S. Marban E. O’Rourke B. (2003). Synchronized whole cell oscillations in mitochondrial metabolism triggered by a local release of reactive oxygen species in cardiac myocytes. *J. Biol. Chem.* 278 44735–44744. 10.1074/jbc.m302673200 12930841

[B10] Aon M. A. Cortassa S. O’Rourke B. (2004). Percolation and criticality in a mitochondrial network. *Proc. Natl. Acad. Sci. U.S.A.* 101 4447–4452. 10.1073/pnas.0307156101 15070738 PMC384767

[B11] Aston D. Capel R. A. Ford K. L. Christian H. C. Mirams G. R. Rog-Zielinska E. A. (2017). High resolution structural evidence suggests the Sarcoplasmic Reticulum forms microdomains with Acidic Stores (lysosomes) in the heart. *Sci. Rep.* 7:40620. 10.1038/srep40620 PMC5240626 28094777

[B12] Bakeeva L. E. Chentsov S. Y. Skulachev V. P. (1978). Mitochondrial framework (reticulum mitochondriale) in rat diaphragm muscle. *Biochim. Biophys. Acta* 501 349–369. 10.1016/0005-2728(78)90104-4 629958

[B13] Bakeeva L. E. Chentsov S. Y. Skulachev V. P. (1983). Intermitochondrial contacts in myocardiocytes. *J. Mol. Cell Cardiol.* 15 413–420. 10.1016/0022-2828(83)90261-4 6620393

[B14] Benador I. Y. Veliova M. Mahdaviani K. Petcherski A. Wikstrom J. D. Assali E. A. (2018). Mitochondria bound to lipid droplets have unique bioenergetics, composition, and dynamics that support lipid droplet expansion. *Cell metabolism* 27 869–885.e6. 29617645 10.1016/j.cmet.2018.03.003 PMC5969538

[B15] Benard G. Bellance N. James D. Parrone P. Fernandez H. Letellier T. (2007). Mitochondrial bioenergetics and structural network organization. *J. Cell Sci.* 120 838–848. 10.1242/jcs.03381 17298981

[B16] Bereiter-Hahn J. (1990). Behavior of mitochondria in the living cell. *Int. Rev. Cytol.* 122 1–63. 10.1016/s0074-7696(08)61205-x 2246114

[B17] Bleazard W. McCaffery J. M. King E. J. Bale S. Mozdy A. Tieu Q. (1999). The dynamin-related GTPase Dnm1 regulates mitochondrial fission in yeast. *Nat. Cell Biol.* 1 298–304. 10.1038/13014 10559943 PMC3739991

[B18] Bleck C. K. E. Kim Y. Willingham T. B. Glancy B. (2018). Subcellular connectomic analyses of energy networks in striated muscle. *Nat. Commun.* 9:5111. 10.1038/s41467-018-07676-y PMC6269443 30504768

[B19] Bochimoto H. Matsuno N. Ishihara Y. Shonaka T. Koga D. Hira Y. (2017). The ultrastructural characteristics of porcine hepatocytes donated after cardiac death and preserved with warm machine perfusion preservation. *PLoS One* 12:e0186352. 10.1371/journal.pone.0186352 29023512 PMC5638504

[B20] Bonnard C. Durand A. Peyrol S. Chanseaume E. Chauvin M. A. Morio B. (2008). Mitochondrial dysfunction results from oxidative stress in the skeletal muscle of diet-induced insulin-resistant mice. *J. Clin. Invest.* 118 789–800. 18188455 10.1172/JCI32601 PMC2176186

[B21] Bosma M. Minnaard R. Sparks L. M. Schaart G. Losen M. de Baets M. H. (2012). The lipid droplet coat protein perilipin 5 also localizes to muscle mitochondria. *Histochem. Cell Biol.* 137 205–216. 10.1007/s00418-011-0888-x 22127648 PMC3262136

[B22] Bosma M. Sparks L. M. Hooiveld G. J. Jorgensen J. A. Houten S. M. Schrauwen P. (2013). Overexpression of PLIN5 in skeletal muscle promotes oxidative gene expression and intramyocellular lipid content without compromising insulin sensitivity. *Biochim. Biophys. Acta* 1831 844–852. 10.1016/j.bbalip.2013.01.007 23353597

[B23] Brand M. D. (2016). Mitochondrial generation of superoxide and hydrogen peroxide as the source of mitochondrial redox signaling. *Free Radic. Biol. Med.* 100 14–31. 10.1016/j.freeradbiomed.2016.04.001 27085844

[B24] Brandt J. T. Martin A. P. Lucas F. V. Vorbeck M. L. (1974). The structure of rat liver mitochondria: a reevaluation. *Biochem. Biophys. Res. Commun.* 59 1097–1104. 10.1016/s0006-291x(74)80091-4 4138008

[B25] Brandt T. Mourier A. Tain L. S. Partridge L. Larsson N. G. Kuhlbrandt W. (2017). Changes of mitochondrial ultrastructure and function during ageing in mice and Drosophila. *eLife* 6:e24662. 10.7554/eLife.24662 PMC5580880 28699890

[B26] Bravo R. Vicencio J. M. Parra V. Troncoso R. Munoz J. P. Bui M. (2011). Increased ER-mitochondrial coupling promotes mitochondrial respiration and bioenergetics during early phases of ER stress. *J. Cell Sci.* 124 2143–2152. 10.1242/jcs.080762 21628424 PMC3113668

[B27] Braymer J. J. Lill R. (2017). Iron-sulfur cluster biogenesis and trafficking in mitochondria. *J. Biol. Chem.* 292:586. 10.1074/jbc.R117.787101 PMC5546016 28615445

[B28] Bubenzer H. J. (1966). Die dunnen und die dicken muskelfasern des zwerchfells der ratte. *Z Zellforsch Mik Ana* 69 520–550. 10.1007/bf00406300 5973111

[B29] Cabodevilla A. G. Sanchez-Caballero L. Nintou E. Boiadjieva V. G. Picatoste F. Gubern A. (2013). Cell survival during complete nutrient deprivation depends on lipid droplet-fueled beta-oxidation of fatty acids. *J. Biol. Chem.* 288 27777–27788. 10.1074/jbc.m113.466656 23940052 PMC3784694

[B30] Calvo S. E. Clauser K. R. Mootha V. K. (2016). MitoCarta2. 0: an updated inventory of mammalian mitochondrial proteins. *Nucleic Acids Res.* 44 D1251–D1257. 26450961 10.1093/nar/gkv1003 PMC4702768

[B31] Cao Y. Xu C. Ye J. He Q. Zhang X. Jia S. (2019). Miro2 regulates inter-mitochondrial communication in the heart and protects against TAC-induced cardiac dysfunction. *Circ. Res.* 125 728–743. 10.1161/circresaha.119.315432 31455181

[B32] Capaldi R. A. Murray J. Byrne L. Janes M. S. Marusich M. F. (2004). Immunological approaches to the characterization and diagnosis of mitochondrial disease. *Mitochondrion* 4 417–426. 10.1016/j.mito.2004.07.006 16120403

[B33] Cereghetti G. M. Stangherlin A. Martins de Brito O. Chang C. R. Blackstone C. Bernardi P. (2008). Dephosphorylation by calcineurin regulates translocation of Drp1 to mitochondria. *Proc. Natl. Acad. Sci. U.S.A.* 105 15803–15808. 10.1073/pnas.0808249105 18838687 PMC2572940

[B34] Chada S. R. Hollenbeck P. J. (2003). Mitochondrial movement and positioning in axons: the role of growth factor signaling. *J. Exp. Biol.* 206 1985–1992. 10.1242/jeb.00263 12756280

[B35] Chada S. R. Hollenbeck P. J. (2004). Nerve growth factor signaling regulates motility and docking of axonal mitochondria. *Curr. Biol.* 14 1272–1276. 10.1016/j.cub.2004.07.027 15268858

[B36] Chami M. Oules B. Szabadkai G. Tacine R. Rizzuto R. Paterlini-Brechot P. (2008). Role of SERCA1 truncated isoform in the proapoptotic calcium transfer from ER to mitochondria during ER stress. *Mol. Cell* 32 641–651. 10.1016/j.molcel.2008.11.014 19061639 PMC2676567

[B37] Chan D. C. (2012). Fusion and fission: interlinked processes critical for mitochondrial health. *Annu Rev Genet* 46 265–287. 10.1146/annurev-genet-110410-132529 22934639

[B38] Chan D. C. (2019). Mitochondrial dynamics and its involvement in disease. *Ann. Rev. Pathol.* 15 235–259. 10.1146/annurev-pathmechdis-012419-032711 31585519

[B39] Chandel N. S. (2014). Mitochondria as signaling organelles. *BMC Biol.* 12:34. 10.1186/1741-7007-12-34 PMC4035690 24884669

[B40] Chandel N. S. McClintock D. S. Feliciano C. E. Wood T. M. Melendez J. A. Rodriguez A. M. (2000). Reactive oxygen species generated at mitochondrial complex III stabilize hypoxia-inducible factor-1alpha during hypoxia: a mechanism of O2 sensing. *J. Biol. Chem.* 275 25130–25138. 10.1074/jbc.m001914200 10833514

[B41] Chen H. Chan D. C. (2005). Emerging functions of mammalian mitochondrial fusion and fission. *Hum. Mol. Genet. 14 Spec. No.* 2 R283–R289. 10.1093/hmg/ddi270 16244327

[B42] Chen H. Chomyn A. Chan D. C. (2005). Disruption of fusion results in mitochondrial heterogeneity and dysfunction. *J. Biol. Chem.* 280 26185–26192. 10.1074/jbc.m503062200 15899901

[B43] Chen H. C. Vermulst M. Wang Y. E. Chomyn A. Prolla T. A. McCaffery J. M. (2010). Mitochondrial fusion is required for mtdna stability in skeletal muscle and tolerance of mtDNA mutations. *Cell* 141 280–289. 10.1016/j.cell.2010.02.026 20403324 PMC2876819

[B44] Chen Q. Jin C. Shao X. Guan R. Tian Z. Wang C. (2018). Super-resolution tracking of mitochondrial dynamics with an iridium(III) luminophore. *Small* 14:e1802166. 10.1002/smll.201802166 30350549

[B45] Chung J. Torta F. Masai K. Lucast L. Czapla H. Tanner L. B. (2015). Intracellular transport PI4P/phosphatidylserine countertransport at ORP5- and ORP8-mediated ER-plasma membrane contacts. *Science* 349 428–432. 10.1126/science.aab1370 26206935 PMC4638224

[B46] Cid-Castro C. Hernandez-Espinosa D. R. Moran J. (2018). ROS as regulators of mitochondrial dynamics in neurons. *Cell Mol. Neurobiol.* 38 995–1007. 10.1007/s10571-018-0584-7 29687234 PMC11481975

[B47] Cinti S. (2018). *Murine Brown Adipose Tissue, Obesity, Type 2 Diabetes and the Adipose Organ.* Berlin: Springer, 13–79.

[B48] Cioni J. M. Lin J. Q. Holtermann A. V. Koppers M. Jakobs M. A. H. Azizi A. (2019). Late Endosomes Act as mRNA Translation Platforms and Sustain Mitochondria in Axons. *Cell* 176 56–72-e15. 30612743 10.1016/j.cell.2018.11.030 PMC6333918

[B49] Cogliati S. Enriquez J. A. Scorrano L. (2016). Mitochondrial cristae: where beauty meets functionality. *Trends Biochem. Sci.* 41 261–273. 10.1016/j.tibs.2016.01.001 26857402

[B50] Collins T. J. Berridge M. J. Lipp P. Bootman M. D. (2002). Mitochondria are morphologically and functionally heterogeneous within cells. *EMBO J.* 21 1616–1627. 10.1093/emboj/21.7.1616 11927546 PMC125942

[B51] Collins T. J. Bootman M. D. (2003). Mitochondria are morphologically heterogeneous within cells. *J. Exp. Biol.* 206 1993–2000. 10.1242/jeb.00244 12756281

[B52] Cribbs J. T. Strack S. (2007). Reversible phosphorylation of Drp1 by cyclic AMP-dependent protein kinase and calcineurin regulates mitochondrial fission and cell death. *EMBO Rep.* 8 939–944. 10.1038/sj.embor.7401062 17721437 PMC2002551

[B53] Csordas G. Renken C. Varnai P. Walter L. Weaver D. Buttle K. F. (2006). Structural and functional features and significance of the physical linkage between ER and mitochondria. *J. Cell Biol.* 174 915–921. 10.1083/jcb.200604016 16982799 PMC2064383

[B54] Daniele T. Hurbain I. Vago R. Casari G. Raposo G. Tacchetti C. (2014). Mitochondria and melanosomes establish physical contacts modulated by Mfn2 and involved in organelle biogenesis. *Curr. Biol.* 24 393–403. 10.1016/j.cub.2014.01.007 24485836

[B55] Das A. Nag S. Mason A. B. Barroso M. M. (2016). Endosome-mitochondria interactions are modulated by iron release from transferrin. *J. Cell Biol.* 214 831–845. 10.1083/jcb.201602069 27646275 PMC5037410

[B56] Davidowitz J. Philips G. Chiarandini D. J. Breinin G. M. (1984). Intermitochondrial junctions in the extraocular muscle of the rat. *Cell Tissue Res.* 238 417–419. 6509518 10.1007/BF00217317

[B57] Davies K. M. Anselmi C. Wittig I. Faraldo-Gomez J. D. Kuhlbrandt W. (2012). Structure of the yeast F1Fo-ATP synthase dimer and its role in shaping the mitochondrial cristae. *Proc. Natl. Acad. Sci. U.S.A.* 109 13602–13607. 10.1073/pnas.1204593109 22864911 PMC3427116

[B58] de Brito O. M. Scorrano L. (2008). Mitofusin 2 tethers endoplasmic reticulum to mitochondria. *Nature* 456 605–610. 10.1038/nature07534 19052620

[B59] De Vos K. J. Morotz G. M. Stoica R. Tudor E. L. Lau K. F. Ackerley S. (2012). VAPB interacts with the mitochondrial protein PTPIP51 to regulate calcium homeostasis. *Hum. Mol. Genet.* 21 1299–1311. 10.1093/hmg/ddr559 22131369 PMC3284118

[B60] de-Lima-Junior J. C. Souza G. F. Moura-Assis A. Gaspar R. S. Gaspar J. M. Rocha A. L. (2019). Abnormal brown adipose tissue mitochondrial structure and function in IL10 deficiency. *EBioMedicine* 39 436–447. 10.1016/j.ebiom.2018.11.041 30502051 PMC6355943

[B61] Detmer S. A. Chan D. C. (2007). Functions and dysfunctions of mitochondrial dynamics. *Nat. Rev. Mol. Cell Biol.* 8 870–879. 10.1038/nrm2275 17928812

[B62] Ding W. X. Li M. Biazik J. M. Morgan D. G. Guo F. Ni H. M. (2012). Electron microscopic analysis of a spherical mitochondrial structure. *J. Biol. Chem.* 287 42373–42378. 10.1074/jbc.m112.413674 23093403 PMC3516780

[B63] Ding X. Zhang W. Zhao T. Yan C. Du H. (2017). Rab7 GTPase controls lipid metabolic signaling in myeloid-derived suppressor cells. *Oncotarget* 8 30123–30137. 10.18632/oncotarget.16280 28415797 PMC5444731

[B64] Dlasková A. Engstová H. Špaček T. Kahancová A. Pavluch V. Smolková K. (2018). 3D super-resolution microscopy reflects mitochondrial cristae alternations and mtDNA nucleoid size and distribution. *Biochim. Biophys. Acta (BBA) Bioenerget.* 1859 829–844. 10.1016/j.bbabio.2018.04.013 29727614

[B65] Dlasková A. Špaček T. Engstová H. Špačková J. Schröfel A. Holendová B. (2019). Mitochondrial cristae narrowing upon higher 2-oxoglutarate load. *Biochim. Biophys. Acta (BBA) Bioenerget.* 1860 659–678. 10.1016/j.bbabio.2019.06.015 31247171

[B66] Dlasková A. Špaček T. Šantorová J. Plecitá-Hlavatá L. Berková Z. Saudek F. (2010). 4Pi microscopy reveals an impaired three-dimensional mitochondrial network of pancreatic islet β-cells, an experimental model of type-2 diabetes. *Biochim. Biophys. Acta (BBA) Bioenergetics* 1797 1327–1341. 10.1016/j.bbabio.2010.02.003 20144584

[B67] Dorn G. W. (2019). Evolving concepts of mitochondrial dynamics. *Ann. Rev. Physiol.* 81 1–17. 10.1146/annurev-physiol-020518-114358 30256725

[B68] Duvert M. Mazat J. P. Barets A. L. (1985). Intermitochondrial junctions in the heart of the frog, Rana esculenta. A thin-section and freeze-fracture study. *Cell Tissue Res.* 241 129–137. 10.1007/bf00214634 3875412

[B69] Egner A. Jakobs S. Hell S. W. (2002). Fast 100-nm resolution three-dimensional microscope reveals structural plasticity of mitochondria in live yeast. *Proc. Natl. Acad. Sci. U.S.A.* 99 3370–3375. 10.1073/pnas.052545099 11904401 PMC122530

[B70] Eisner V. Cupo R. R. Gao E. Csordas G. Slovinsky W. S. Paillard M. (2017). Mitochondrial fusion dynamics is robust in the heart and depends on calcium oscillations and contractile activity. *Proc. Natl. Acad. Sci. U.S.A.* 114 E859–E868. 28096338 10.1073/pnas.1617288114 PMC5293028

[B71] Eisner V. Lenaers G. Hajnoczky G. (2014). Mitochondrial fusion is frequent in skeletal muscle and supports excitation-contraction coupling. *J. Cell Biol.* 205 179–195. 10.1083/jcb.201312066 24751540 PMC4003250

[B72] Elbaz-Alon Y. Rosenfeld-Gur E. Shinder V. Futerman A. H. Geiger T. Schuldiner M. (2014). interface between vacuoles and mitochondria in yeast. *Dev. Cell* 30 95–102. 10.1016/j.devcel.2014.06.007 25026036

[B73] Favaro G. Romanello V. Varanita T. Andrea Desbats M. Morbidoni V. Tezze C. (2019). DRP1-mediated mitochondrial shape controls calcium homeostasis and muscle mass. *Nat. Commun.* 10:2576. 10.1038/s41467-019-10226-9 PMC6561930 31189900

[B74] Fermie J. Liv N. Ten Brink C. van Donselaar E. G. Muller W. H. Schieber N. L. (2018). Single organelle dynamics linked to 3D structure by correlative live-cell imaging and 3D electron microscopy. *Traffic* 19 354–369. 10.1111/tra.12557 29451726

[B75] Franco A. Kitsis R. N. Fleischer J. A. Gavathiotis E. Kornfeld O. S. Gong G. (2016). Correcting mitochondrial fusion by manipulating mitofusin conformations. *Nature* 540 74–79. 10.1038/nature20156 27775718 PMC5315023

[B76] Franzini-Armstrong C. Protasi F. Ramesh V. (1998). Comparative ultrastructure of Ca2+ release units in skeletal and cardiac muscle. *Ann. N. Y. Acad. Sci.* 853 20–30. 10.1111/j.1749-6632.1998.tb08253.x 10603933

[B77] Franzini-Armstrong C. Protasi F. Tijskens P. (2005). The assembly of calcium release units in cardiac muscle. *Ann. N. Y. Acad. Sci.* 1047 76–85. 10.1196/annals.1341.007 16093486

[B78] Frey T. G. Mannella C. A. (2000). The internal structure of mitochondria. *Trends Biochem. Sci.* 25 319–324. 10.1016/s0968-0004(00)01609-1 10871882

[B79] Frezza C. (2017). Mitochondrial metabolites: undercover signalling molecules. *Interface Focus* 7:20160100. 10.1098/rsfs.2016.0100 28382199 PMC5311903

[B80] Frezza C. Cipolat S. Martins de Brito O. Micaroni M. Beznoussenko G. V. Rudka T. (2006). OPA1 controls apoptotic cristae remodeling independently from mitochondrial fusion. *Cell* 126 177–189. 10.1016/j.cell.2006.06.025 16839885

[B81] Friedman J. R. Lackner L. L. West M. DiBenedetto J. R. Nunnari J. Voeltz G. K. (2011). ER tubules mark sites of mitochondrial division. *Science* 334 358–362. 10.1126/science.1207385 21885730 PMC3366560

[B82] Friedman J. R. Nunnari J. (2014). Mitochondrial form and function. *Nature* 505 335–343. 10.1038/nature12985 24429632 PMC4075653

[B83] Friedman J. R. Webster B. M. Mastronarde D. N. Verhey K. J. Voeltz G. K. (2010). ER sliding dynamics and ER-mitochondrial contacts occur on acetylated microtubules. *J. Cell Biol.* 190 363–375. 10.1083/jcb.200911024 20696706 PMC2922647

[B84] Galmes R. Houcine A. van Vliet A. R. Agostinis P. Jackson C. L. Giordano F. (2016). ORP5/ORP8 localize to endoplasmic reticulum-mitochondria contacts and are involved in mitochondrial function. *EMBO Rep.* 17 800–810. 10.15252/embr.201541108 27113756 PMC5278607

[B85] Gao R. Asano S. M. Upadhyayula S. Pisarev I. Milkie D. E. Liu T. L. (2019). Cortical column and whole-brain imaging with molecular contrast and nanoscale resolution. *Science* 363:eaau8302. 10.1126/science.aau8302 30655415 PMC6481610

[B86] Gilkerson R. W. Selker J. M. Capaldi R. A. (2003). The cristal membrane of mitochondria is the principal site of oxidative phosphorylation. *FEBS Lett.* 546 355–358. 10.1016/s0014-5793(03)00633-1 12832068

[B87] Giorgi C. Marchi S. Pinton P. (2018). The machineries, regulation and cellular functions of mitochondrial calcium. *Nat. Rev. Mol. Cell Biol.* 19 713–730. 10.1038/s41580-018-0052-8 30143745

[B88] Glancy B. (2020). Visualizing Mitochondrial Form and Function within the Cell. *Trends Mol. Med.* 26 58–70. 10.1016/j.molmed.2019.09.009 31706841 PMC6938546

[B89] Glancy B. Hartnell L. M. Combs C. A. Fenmou A. Sun J. Murphy E. (2017). Power grid protection of the muscle mitochondrial reticulum. *Cell Rep.* 19 487–496. 10.1016/j.celrep.2017.03.063 28423313 PMC5490369

[B90] Glancy B. Hartnell L. M. Malide D. Yu Z. X. Combs C. A. Connelly P. S. (2015). Mitochondrial reticulum for cellular energy distribution in muscle. *Nature* 523 617–620. 10.1038/nature14614 26223627 PMC6988728

[B91] Glancy B. Willis W. T. Chess D. J. Balaban R. S. (2013). Effect of calcium on the oxidative phosphorylation cascade in skeletal muscle mitochondria. *Biochemistry* 52 2793–2809. 10.1021/bi3015983 23547908 PMC4157357

[B92] Glater E. E. Megeath L. J. Stowers R. S. Schwarz T. L. (2006). Axonal transport of mitochondria requires milton to recruit kinesin heavy chain and is light chain independent. *J. Cell Biol.* 173 545–557. 10.1083/jcb.200601067 16717129 PMC2063864

[B93] Gomes L. C. Di Benedetto G. Scorrano L. (2011). During autophagy mitochondria elongate, are spared from degradation and sustain cell viability. *Nat. Cell Biol.* 13 589–598. 10.1038/ncb2220 21478857 PMC3088644

[B94] Gomes L. C. Scorrano L. (2011). Mitochondrial elongation during autophagy: a stereotypical response to survive in difficult times. *Autophagy* 7 1251–1253. 10.4161/auto.7.10.16771 21743300 PMC3242616

[B95] Gomez-Suaga P. Paillusson S. Stoica R. Noble W. Hanger D. P. Miller C. C. J. (2017). The ER-Mitochondria Tethering Complex VAPB-PTPIP51 Regulates Autophagy. *Curr. Biol.* 27 371–385. 10.1016/j.cub.2016.12.038 28132811 PMC5300905

[B96] Granneman J. G. Moore H. P. Mottillo E. P. Zhu Z. Zhou L. (2011). Interactions of perilipin-5 (Plin5) with adipose triglyceride lipase. *J. Biol. Chem.* 286 5126–5135. 10.1074/jbc.m110.180711 21148142 PMC3037624

[B97] Guo X. Macleod G. T. Wellington A. Hu F. Panchumarthi S. Schoenfield M. (2005). The GTPase dMiro is required for axonal transport of mitochondria to Drosophila synapses. *Neuron* 47 379–393. 10.1016/j.neuron.2005.06.027 16055062

[B98] Guo Y. Li D. Zhang S. Yang Y. Liu J. J. Wang X. (2018). Visualizing intracellular organelle and cytoskeletal interactions at nanoscale resolution on millisecond timescales. *Cell* 1 430–1442.e17. 10.1016/j.cell.2018.09.057 30454650

[B99] Hackenbrock C. R. (1966). Ultrastructural bases for metabolically linked mechanical activity in mitochondria. I. Reversible ultrastructural changes with change in metabolic steady state in isolated liver mitochondria. *J. Cell Biol.* 30 269–297. 10.1083/jcb.30.2.269 5968972 PMC2107001

[B100] Hailey D. W. Rambold A. S. Satpute-Krishnan P. Mitra K. Sougrat R. Kim P. K. (2010). Mitochondria supply membranes for autophagosome biogenesis during starvation. *Cell* 141 656–667. 10.1016/j.cell.2010.04.009 20478256 PMC3059894

[B101] Hajnoczky G. Hager R. Thomas A. P. (1999). Mitochondria suppress local feedback activation of inositol 1,4, 5-trisphosphate receptors by Ca2+. *J. Biol. Chem.* 274 14157–14162. 10.1074/jbc.274.20.14157 10318833

[B102] Hamdi A. Roshan T. M. Kahawita T. M. Mason A. B. Sheftel A. D. Ponka P. (2016). Erythroid cell mitochondria receive endosomal iron by a “kiss-and-run” mechanism. *Biochim. Biophys. Acta* 1863 2859–2867. 10.1016/j.bbamcr.2016.09.008 27627839

[B103] Han Y. Li M. Qiu F. Zhang M. Zhang Y. H. (2017). Cell-permeable organic fluorescent probes for live-cell long-term super-resolution imaging reveal lysosome-mitochondrion interactions. *Nat. Commun.* 8:1307. 10.1038/s41467-017-01503-6 PMC5670236 29101340

[B104] Hara Y. Yuk F. Puri R. Janssen W. G. Rapp P. R. Morrison J. H. (2014). Presynaptic mitochondrial morphology in monkey prefrontal cortex correlates with working memory and is improved with estrogen treatment. *Proc. Natl. Acad. Sci. U.S.A.* 111 486–491. 10.1073/pnas.1311310110 24297907 PMC3890848

[B105] Harmon H. J. Hall J. D. Crane F. L. (1974). Structure of mitochondrial cristae membranes. *Biochim. Biophys. Acta* 344 119–155. 10.1016/0304-4157(74)90002-1 4153673

[B106] Harner M. Korner C. Walther D. Mokranjac D. Kaesmacher J. Welsch U. (2011). The mitochondrial contact site complex, a determinant of mitochondrial architecture. *EMBO J.* 30 4356–4370. 10.1038/emboj.2011.379 22009199 PMC3230385

[B107] Hennings T. G. Chopra D. G. DeLeon E. R. VanDeusen H. R. Sesaki H. Merrins M. J. (2018). In vivo deletion of beta-cell drp1 impairs insulin secretion without affecting islet oxygen consumption. *Endocrinology* 159 3245–3256. 10.1210/en.2018-00445 30052866 PMC6107751

[B108] Hermann G. J. Shaw J. M. (1998). Mitochondrial dynamics in yeast. *Annu. Rev. Cell Dev. Biol.* 14 265–303. 9891785 10.1146/annurev.cellbio.14.1.265

[B109] Herms A. Bosch M. Reddy B. J. Schieber N. L. Fajardo A. Ruperez C. (2015). AMPK activation promotes lipid droplet dispersion on detyrosinated microtubules to increase mitochondrial fatty acid oxidation. *Nat. Commun.* 6:7176. 10.1038/ncomms8176 PMC4446796 26013497

[B110] Honscher C. Mari M. Auffarth K. Bohnert M. Griffith J. Geerts W. (2014). Cellular metabolism regulates contact sites between vacuoles and mitochondria. *Dev. Cell* 30 86–94. 10.1016/j.devcel.2014.06.006 25026035

[B111] Hoppel C. L. Tandler B. Fujioka H. Riva A. (2009). Dynamic organization of mitochondria in human heart and in myocardial disease. *Int. J. Biochem. Cell Biol.* 41 1949–1956. 10.1016/j.biocel.2009.05.004 19446651 PMC3221317

[B112] Hu C. K. Coughlin M. Field C. M. Mitchison T. J. (2008). Cell polarization during monopolar cytokinesis. *J. Cell Biol.* 181 195–202. 10.1083/jcb.200711105 18411311 PMC2315668

[B113] Huang X. Fan J. Li L. Liu H. Wu R. Wu Y. (2018). Fast, long-term, super-resolution imaging with Hessian structured illumination microscopy. *Nat. Biotechnol.* 36 451–459. 10.1038/nbt.4115 29644998

[B114] Huang X. Sun L. Ji S. Zhao T. Zhang W. Xu J. (2013). Kissing and nanotunneling mediate intermitochondrial communication in the heart. *Proc. Natl. Acad. Sci. U.S.A.* 110 2846–2851. 10.1073/pnas.1300741110 23386722 PMC3581932

[B115] Ikon N. Ryan R. O. (2017). Cardiolipin and mitochondrial cristae organization. *Biochim. Biophys. Acta (BBA) Biomembranes* 1859 1156–1163. 10.1016/j.bbamem.2017.03.013 28336315 PMC5426559

[B116] Inoué T. Koike H. (1989). High−resolution low−temperature scanning electron microscopy for observing intracellular structures of quick frozen biological specimens. *J. Microsc.* 156 137–147. 10.1111/j.1365-2818.1989.tb02913.x 2593147

[B117] Ishihara N. Fujita Y. Oka T. Mihara K. (2006). Regulation of mitochondrial morphology through proteolytic cleavage of OPA1. *EMBO J.* 25 2966–2977. 10.1038/sj.emboj.7601184 16778770 PMC1500981

[B118] Iwasawa R. Mahul-Mellier A. L. Datler C. Pazarentzos E. Grimm S. (2011). Fis1 and Bap31 bridge the mitochondria-ER interface to establish a platform for apoptosis induction. *EMBO J.* 30 556–568. 10.1038/emboj.2010.346 21183955 PMC3034017

[B119] Jakobs S. Stephan T. Ilgen P. Brüser C. (2020). Light microscopy of mitochondria at the nanoscale. *Annu. Rev. Biophys.* 49 289–308. 10.1146/annurev-biophys-121219-081550 32092283 PMC7610798

[B120] Ježek P. Dlasková A. (2019). Dynamic of mitochondrial network, cristae, and mitochondrial nucleoids in pancreatic β-cells. *Mitochondrion* 49 245–258. 10.1016/j.mito.2019.06.007 31252091

[B121] John Peter A. T. Herrmann B. Antunes D. Rapaport D. Dimmer K. S. Kornmann B. (2017). Vps13-Mcp1 interact at vacuole-mitochondria interfaces and bypass ER-mitochondria contact sites. *J. Cell Biol.* 216 3219–3229. 10.1083/jcb.201610055 28864540 PMC5626531

[B122] Johnson D. T. Harris R. A. French S. Blair P. V. You J. Bemis K. G. (2007). Tissue heterogeneity of the mammalian mitochondrial proteome. *Am. J. Physiol. Cell Physiol.* 292 C689–C697. 16928776 10.1152/ajpcell.00108.2006

[B123] Juhaszova M. Zorov D. B. Kim S. H. Pepe S. Fu Q. Fishbein K. W. (2004). Glycogen synthase kinase-3beta mediates convergence of protection signaling to inhibit the mitochondrial permeability transition pore. *J. Clin. Invest.* 113 1535–1549. 10.1172/jci19906 15173880 PMC419483

[B124] Kanamura S. Kanai K. Oka M. Shugyo Y. Watanabe J. (1985). Quantitative analysis of development of mitochondrial ultrastructure in differentiating mouse hepatocytes during postnatal period. *J. Ultrastruct. Res.* 93 195–204. 10.1016/0889-1605(85)90099-0 3837129

[B125] Kang H. W. Ribich S. Kim B. W. Hagen S. J. Bianco A. C. Cohen D. E. (2009). Mice lacking Pctp/StarD2 exhibit increased adaptive thermogenesis and enlarged mitochondria in brown adipose tissue. *J. Lipid. Res.* 50 2212–2221. 10.1194/jlr.m900013-jlr200 19502644 PMC2759827

[B126] Kanzaki Y. Terasaki F. Okabe M. Otsuka K. Katashima T. Fujita S. (2010). Giant mitochondria in the myocardium of a patient with mitochondrial cardiomyopathy: transmission and 3-dimensional scanning electron microscopy. *Circulation* 121 831–832. 10.1161/cir.0b013e3181d22e2d 20159843

[B127] Karbowski M. Arnoult D. Chen H. Chan D. C. Smith C. L. Youle R. J. (2004). Quantitation of mitochondrial dynamics by photolabeling of individual organelles shows that mitochondrial fusion is blocked during the Bax activation phase of apoptosis. *J. Cell Biol.* 164 493–499. 10.1083/jcb.200309082 14769861 PMC2172000

[B128] Karbowski M. Youle R. J. (2003). Dynamics of mitochondrial morphology in healthy cells and during apoptosis. *Cell Death Differ.* 10 870–880. 10.1038/sj.cdd.4401260 12867994

[B129] Kirkwood S. P. Munn E. A. Brooks G. A. (1986). Mitochondrial reticulum in limb skeletal muscle. *Am. J. Physiol.* 251 C395–C402. 3752235 10.1152/ajpcell.1986.251.3.C395

[B130] Klecker T. Scholz D. Fortsch J. Westermann B. (2013). The yeast cell cortical protein Num1 integrates mitochondrial dynamics into cellular architecture. *J. Cell Sci.* 126 2924–2930. 10.1242/jcs.126045 23641071

[B131] Kondadi A. K. Anand R. Hansch S. Urbach J. Zobel T. Wolf D. M. (2020). Cristae undergo continuous cycles of membrane remodelling in a MICOS-dependent manner. *EMBO Rep.* 21:e49776. 10.15252/embr.201949776 PMC7054676 32067344

[B132] Koopman W. J. Visch H. J. Verkaart S. van den Heuvel L. W. Smeitink J. A. Willems P. H. (2005). Mitochondrial network complexity and pathological decrease in complex I activity are tightly correlated in isolated human complex I deficiency. *Am. J. Physiol. Cell Physiol.* 289 C881–C890. 15901599 10.1152/ajpcell.00104.2005

[B133] Kornmann B. Currie E. Collins S. R. Schuldiner M. Nunnari J. Weissman J. S. (2009). An ER-mitochondria tethering complex revealed by a synthetic biology screen. *Science* 325 477–481. 10.1126/science.1175088 19556461 PMC2933203

[B134] Kukat C. Davies K. M. Wurm C. A. Spahr H. Bonekamp N. A. Kuhl I. (2015). Cross-strand binding of TFAM to a single mtDNA molecule forms the mitochondrial nucleoid. *Proc. Natl. Acad. Sci. U.S.A.* 112 11288–11293. 10.1073/pnas.1512131112 26305956 PMC4568684

[B135] Kurz F. T. Aon M. A. O’Rourke B. Armoundas A. A. (2010). Spatio-temporal oscillations of individual mitochondria in cardiac myocytes reveal modulation of synchronized mitochondrial clusters. *Proc. Natl. Acad. Sci. U.S.A.* 107 14315–14320. 10.1073/pnas.1007562107 20656937 PMC2922526

[B136] Kuznetsov A. V. Schneeberger S. Renz O. Meusburger H. Saks V. Usson Y. (2004a). Functional heterogeneity of mitochondria after cardiac cold ischemia and reperfusion revealed by confocal imaging. *Transplantation* 77 754–756. 10.1097/01.tp.0000115346.85679.34 15021841

[B137] Kuznetsov A. V. Troppmair J. Sucher R. Hermann M. Saks V. Margreiter R. (2006). Mitochondrial subpopulations and heterogeneity revealed by confocal imaging: possible physiological role? *Biochim. Biophys. Acta* 1757 686–691. 10.1016/j.bbabio.2006.03.014 16712778

[B138] Kuznetsov A. V. Usson Y. Leverve X. Margreiter R. (2004b). Subcellular heterogeneity of mitochondrial function and dysfunction: evidence obtained by confocal imaging. *Mol. Cell Biochem.* 257 359–365. 10.1023/b:mcbi.0000009881.01943.68 14977194

[B139] Kuznetsov A. V. Winkler K. Wiedemann F. R. von Bossanyi P. Dietzmann K. Kunz W. S. (1998). Impaired mitochondrial oxidative phosphorylation in skeletal muscle of the dystrophin-deficient mdx mouse. *Mol. Cell Biochem.* 183 87–96. 9655182 10.1023/a:1006868130002

[B140] Labbe K. Murley A. Nunnari J. (2014). Determinants and functions of mitochondrial behavior. *Annu. Rev. Cell Dev. Biol.* 30 357–391. 10.1146/annurev-cellbio-101011-155756 25288115

[B141] Lackner L. L. Ping H. Graef M. Murley A. Nunnari J. (2013). Endoplasmic reticulum-associated mitochondria-cortex tether functions in the distribution and inheritance of mitochondria. *Proc. Natl. Acad. Sci. U.S.A.* 110 E458–E467. 23341591 10.1073/pnas.1215232110 PMC3568303

[B142] Lavorato M. Iyer V. R. Dewight W. Cupo R. R. Debattisti V. Gomez L. (2017). Increased mitochondrial nanotunneling activity, induced by calcium imbalance, affects intermitochondrial matrix exchanges. *Proc. Natl. Acad. Sci. U.S.A.* 114 E849–E858. 28096415 10.1073/pnas.1617788113 PMC5293110

[B143] Lawrence E. J. Mandato C. A. (2013). Mitochondria localize to the cleavage furrow in mammalian cytokinesis. *PLoS One* 8:e72886. 10.1371/journal.pone.0072886 23991162 PMC3749163

[B144] Lawrie A. M. Rizzuto R. Pozzan T. Simpson A. W. (1996). A role for calcium influx in the regulation of mitochondrial calcium in endothelial cells. *J. Biol. Chem.* 271 10753–10759. 10.1074/jbc.271.18.10753 8631885

[B145] Leduc-Gaudet J. P. Picard M. Pelletier F. S. Sgarioto N. Auger M. J. Vallee J. (2015). Mitochondrial morphology is altered in atrophied skeletal muscle of aged mice. *Oncotarget* 6 17923–17937. 10.18632/oncotarget.4235 26053100 PMC4627226

[B146] Lever J. Chappell J. (1958). Mitochondria isolated from rat brown adipose tissue and liver. *J. Cell Biol.* 4 287–290. 10.1083/jcb.4.3.287 13549500 PMC2224483

[B147] Lewis S. C. Uchiyama L. F. Nunnari J. (2016). ER-mitochondria contacts couple mtDNA synthesis with mitochondrial division in human cells. *Science* 353:aaf5549. 10.1126/science.aaf5549 27418514 PMC5554545

[B148] Ligon L. A. Steward O. (2000). Role of microtubules and actin filaments in the movement of mitochondria in the axons and dendrites of cultured hippocampal neurons. *J. Comp. Neurol.* 427 351–361. 10.1002/1096-9861(20001120)427:3<351::aid-cne3>3.0.co;2-r 11054698

[B149] Liu X. Hajnoczky G. (2011). Altered fusion dynamics underlie unique morphological changes in mitochondria during hypoxia-reoxygenation stress. *Cell Death Different.* 18 1561–1572. 10.1038/cdd.2011.13 21372848 PMC3172112

[B150] Lodi R. Tonon C. Valentino M. L. Iotti S. Clementi V. Malucelli E. (2004). Deficit of in vivo mitochondrial ATP production in OPA1-related dominant optic atrophy. *Ann. Neurol.* 56 719–723. 10.1002/ana.20278 15505825

[B151] Long Q. Zhao D. Fan W. Yang L. Zhou Y. Qi J. (2015). Modeling of mitochondrial donut formation. *Biophys. J.* 109 892–899. 10.1016/j.bpj.2015.07.039 26331247 PMC4564827

[B152] Lopez-Otin C. Blasco M. A. Partridge L. Serrano M. Kroemer G. (2013). The hallmarks of aging. *Cell* 153 1194–1217. 23746838 10.1016/j.cell.2013.05.039 PMC3836174

[B153] Mannella C. A. (2006). The relevance of mitochondrial membrane topology to mitochondrial function. *Biochim. Biophys. Acta (BBA) Mol. Basis Dis.* 1762 140–147. 10.1016/j.bbadis.2005.07.001 16054341

[B154] Mannella C. A. Lederer W. J. Jafri M. S. (2013). The connection between inner membrane topology and mitochondrial function. *J. Mol. Cell Cardiol.* 62 51–57. 10.1016/j.yjmcc.2013.05.001 23672826 PMC4219563

[B155] Martínez-Reyes I. Chandel N. S. (2020). Mitochondrial TCA cycle metabolites control physiology and disease. *Nat. Commun.* 11 1–11. 31900386 10.1038/s41467-019-13668-3 PMC6941980

[B156] McCarron J. G. Olson M. L. Wilson C. Sandison M. E. Chalmers S. (2013). Examining the role of mitochondria in Ca(2)(+) signaling in native vascular smooth muscle. *Microcirculation* 20 317–329. 10.1111/micc.12039 23305516 PMC3708117

[B157] Miller C. R. Waits L. P. Joyce P. (2006). Phylogeography and mitochondrial diversity of extirpated brown bear (*Ursus arctos*) populations in the contiguous United States and Mexico. *Mol. Ecol.* 15 4477–4485. 10.1111/j.1365-294x.2006.03097.x 17107477

[B158] Miller K. E. Sheetz M. P. (2004). Axonal mitochondrial transport and potential are correlated. *J. Cell Sci.* 117 2791–2804. 10.1242/jcs.01130 15150321

[B159] Misgeld T. Kerschensteiner M. Bareyre F. M. Burgess R. W. Lichtman J. W. (2007). Imaging axonal transport of mitochondria in vivo. *Nat. Methods* 4 559–561. 10.1038/nmeth1055 17558414

[B160] Miyazono Y. Hirashima S. Ishihara N. Kusukawa J. Nakamura K. I. Ohta K. (2018). Uncoupled mitochondria quickly shorten along their long axis to form indented spheroids, instead of rings, in a fission-independent manner. *Sci. Rep.* 8:350. 10.1038/s41598-017-18582-6 PMC5762872 29321618

[B161] Molina A. J. Wikstrom J. D. Stiles L. Las G. Mohamed H. Elorza A. (2009). Mitochondrial networking protects beta-cells from nutrient-induced apoptosis. *Diabetes* 58 2303–2315. 10.2337/db07-1781 19581419 PMC2750232

[B162] Mootha V. K. Bunkenborg J. Olsen J. V. Hjerrild M. Wisniewski J. R. Stahl E. (2003). Integrated analysis of protein composition, tissue diversity, and gene regulation in mouse mitochondria. *Cell* 115 629–640. 10.1016/s0092-8674(03)00926-7 14651853

[B163] Morre D. J. Merritt W. D. Lembi C. A. (1971). Connections between mitochondria and endoplasmic reticulum in rat liver and onion stem. *Protoplasma* 73 43–49. 10.1007/bf01286410 5112775

[B164] Morris R. L. Hollenbeck P. J. (1993). The regulation of bidirectional mitochondrial transport is coordinated with axonal outgrowth. *J. Cell Sci.* 104(Pt 3) 917–927. 8314882 10.1242/jcs.104.3.917

[B165] Morris R. L. Hollenbeck P. J. (1995). Axonal transport of mitochondria along microtubules and F-actin in living vertebrate neurons. *J. Cell Biol.* 131 1315–1326. 10.1083/jcb.131.5.1315 8522592 PMC2120647

[B166] Mühleip A. W. Joos F. Wigge C. Frangakis A. S. Kühlbrandt W. Davies K. M. (2016). Helical arrays of U-shaped ATP synthase dimers form tubular cristae in ciliate mitochondria. *Proc. Natl. Acad. Sci. U.S.A.* 113 8442–8447. 10.1073/pnas.1525430113 27402755 PMC4968746

[B167] Munoz-Braceras S. Calvo R. Escalante R. (2015). TipC and the chorea-acanthocytosis protein VPS13A regulate autophagy in Dictyostelium and human HeLa cells. *Autophagy* 11 918–927. 10.1080/15548627.2015.1034413 25996471 PMC4507429

[B168] Munoz-Braceras S. Tornero-Ecija A. R. Vincent O. Escalante R. (2019). VPS13A is closely associated with mitochondria and is required for efficient lysosomal degradation. *Dis. Model. Mech.* 12:dmm036681. 10.1242/dmm.036681 30709847 PMC6398486

[B169] Murphy A. N. Kelleher J. K. Fiskum G. (1990). Submicromolar Ca2+ regulates phosphorylating respiration by normal rat liver and AS-30D hepatoma mitochondria by different mechanisms. *J. Biol. Chem.* 265 10527–10534. 2113059

[B170] Nemani N. Carvalho E. Tomar D. Dong Z. Ketschek A. Breves S. L. (2018). MIRO-1 Determines Mitochondrial Shape Transition upon GPCR Activation and Ca(2+) Stress. *Cell Rep.* 23 1005–1019. 10.1016/j.celrep.2018.03.098 29694881 PMC5973819

[B171] Nguyen T. B. Louie S. M. Daniele J. R. Tran Q. Dillin A. Zoncu R. (2017). DGAT1-dependent lipid droplet biogenesis protects mitochondrial function during starvation-induced autophagy. *Dev Cell* 42 9–21.e5. 28697336 10.1016/j.devcel.2017.06.003 PMC5553613

[B172] Nguyen T. T. Oh S. S. Weaver D. Lewandowska A. Maxfield D. Schuler M. H. (2014). Loss of Miro1-directed mitochondrial movement results in a novel murine model for neuron disease. *Proc. Natl. Acad. Sci. U.S.A.* 111 E3631–E3640. 25136135 10.1073/pnas.1402449111 PMC4156725

[B173] Nixon G. F. Mignery G. A. Somlyo A. V. (1994). Immunogold localization of inositol 1,4,5-trisphosphate receptors and characterization of ultrastructural features of the sarcoplasmic reticulum in phasic and tonic smooth muscle. *J. Muscle Res. Cell Motil.* 15 682–700. 10.1007/bf00121075 7706424

[B174] Nixon-Abell J. Obara C. J. Weigel A. V. Li D. Legant W. R. Xu C. S. (2016). Increased spatiotemporal resolution reveals highly dynamic dense tubular matrices in the peripheral ER. *Science* 354:aaf3928. 10.1126/science.aaf3928 27789813 PMC6528812

[B175] Nunnari J. Marshall W. F. Straight A. Murray A. Sedat J. W. Walter P. (1997). Mitochondrial transmission during mating in Saccharomyces cerevisiae is determined by mitochondrial fusion and fission and the intramitochondrial segregation of mitochondrial DNA. *Mol. Biol. Cell* 8 1233–1242. 10.1091/mbc.8.7.1233 9243504 PMC276149

[B176] Ogata T. Yamasaki Y. (1985). Scanning electron-microscopic studies on the three-dimensional structure of mitochondria in the mammalian red, white and intermediate muscle fibers. *Cell Tissue Res.* 241 251–256. 10.1007/bf00217168 4028126

[B177] Ohlmeier S. Kastaniotis A. J. Hiltunen J. K. Bergmann U. (2004). The yeast mitochondrial proteome, a study of fermentative and respiratory growth. *J. Biol. Chem.* 279 3956–3979. 10.1074/jbc.m310160200 14597615

[B178] Olichon A. Emorine L. J. Descoins E. Pelloquin L. Brichese L. Gas N. (2002). The human dynamin-related protein OPA1 is anchored to the mitochondrial inner membrane facing the inter-membrane space. *FEBS Lett.* 523 171–176. 10.1016/s0014-5793(02)02985-x 12123827

[B179] Onoue K. Jofuku A. Ban-Ishihara R. Ishihara T. Maeda M. Koshiba T. (2013). Fis1 acts as a mitochondrial recruitment factor for TBC1D15 that is involved in regulation of mitochondrial morphology. *J. Cell Sci.* 126 176–185. 10.1242/jcs.111211 23077178

[B180] Pagliarini D. J. Calvo S. E. Chang B. Sheth S. A. Vafai S. B. Ong S.-E. (2008). A mitochondrial protein compendium elucidates complex I disease biology. *Cell* 134 112–123. 10.1016/j.cell.2008.06.016 18614015 PMC2778844

[B181] Palade G. E. (1953). An electron microscope study of the mitochondrial structure. *J. Histochem. Cytochem.* 1 188–211. 10.1177/1.4.188 13069686

[B182] Park M. K. Ashby M. C. Erdemli G. Petersen O. H. Tepikin A. V. (2001). Perinuclear, perigranular and sub-plasmalemmal mitochondria have distinct functions in the regulation of cellular calcium transport. *EMBO J.* 20 1863–1874. 10.1093/emboj/20.8.1863 11296220 PMC125431

[B183] Patel K. D. Glancy B. Balaban R. S. (2016). The electrochemical transmission in I-Band segments of the mitochondrial reticulum. *Biochim. Biophys. Acta* 1857 1284–1289. 10.1016/j.bbabio.2016.02.014 26921810 PMC4893892

[B184] Pelloux S. Robillard J. Ferrera R. Bilbaut A. Ojeda C. Saks V. (2006). Non-beating HL-1 cells for confocal microscopy: application to mitochondrial functions during cardiac preconditioning. *Prog. Biophys. Mol. Biol.* 90 270–298. 10.1016/j.pbiomolbio.2005.06.009 16140363

[B185] Perkins G. Song J. Tarsa L. Deerinck T. Ellisman M. Frey T. (1998). Electron tomography of mitochondria from brown adipocytes reveals crista junctions. *J. Bioenerg. Biomemb.* 30 431–442. 10.1023/a:1020586012561 9932646

[B186] Pfluger P. T. Kabra D. G. Aichler M. Schriever S. C. Pfuhlmann K. Garcia V. C. (2015). Calcineurin Links Mitochondrial Elongation with Energy Metabolism. *Cell Metab.* 22 838–850. 10.1016/j.cmet.2015.08.022 26411342

[B187] Phillips D. Covian R. Aponte A. M. Glancy B. Taylor J. F. Chess D. (2012). Regulation of oxidative phosphorylation complex activity: effects of tissue-specific metabolic stress within an allometric series and acute changes in workload. *Am. J. Physiol. Regul. Integr. Comp. Physiol.* 302 R1034–R1048. 22378775 10.1152/ajpregu.00596.2011 PMC3362144

[B188] Picard M. McManus M. J. Csordas G. Varnai P. Dorn G. W. II Williams D. (2015). Trans-mitochondrial coordination of cristae at regulated membrane junctions. *Nat. Commun.* 6:6259. 10.1038/ncomms7259 PMC4332397 25687472

[B189] Pich S. Bach D. Briones P. Liesa M. Camps M. Testar X. (2005). The Charcot-Marie-Tooth type 2A gene product, Mfn2, up-regulates fuel oxidation through expression of OXPHOS system. *Hum. Mol. Genet.* 14 1405–1415. 10.1093/hmg/ddi149 15829499

[B190] Pickrell A. M. Youle R. J. (2015). The roles of PINK1, parkin, and mitochondrial fidelity in Parkinson’s disease. *Neuron* 85 257–273. 10.1016/j.neuron.2014.12.007 25611507 PMC4764997

[B191] Pilling A. D. Horiuchi D. Lively C. M. Saxton W. M. (2006). Kinesin-1 and Dynein are the primary motors for fast transport of mitochondria in *Drosophila* motor axons. *Mol. Biol. Cell* 17 2057–2068. 10.1091/mbc.e05-06-0526 16467387 PMC1415296

[B192] Pinton P. Ferrari D. Rapizzi E. Di Virgilio F. Pozzan T. Rizzuto R. (2001). The Ca2+ concentration of the endoplasmic reticulum is a key determinant of ceramide-induced apoptosis: significance for the molecular mechanism of Bcl-2 action. *EMBO J.* 20 2690–2701. 10.1093/emboj/20.11.2690 11387204 PMC125256

[B193] Porat-Shliom N. Harding O. J. Malec L. Narayan K. Weigert R. (2019). Mitochondrial populations exhibit differential dynamic responses to increased energy demand during exocytosis in vivo. *iScience* 11 440–449. 10.1016/j.isci.2018.12.036 30661001 PMC6355620

[B194] Qin J. Guo Y. Xue B. Shi P. Chen Y. Su Q. P. (2020). ER-mitochondria contacts promote mtDNA nucleoids active transportation via mitochondrial dynamic tubulation. *Nat. Commun.* 11 1–12. 32901010 10.1038/s41467-020-18202-4 PMC7478960

[B195] Quintana A. Schwarz E. C. Schwindling C. Lipp P. Kaestner L. Hoth M. (2006). Sustained activity of calcium release-activated calcium channels requires translocation of mitochondria to the plasma membrane. *J. Biol. Chem.* 281 40302–40309. 10.1074/jbc.m607896200 17056596

[B196] Quintana A. Schwindling C. Wenning A. S. Becherer U. Rettig J. Schwarz E. C. (2007). T cell activation requires mitochondrial translocation to the immunological synapse. *Proc. Natl. Acad. Sci. U.S.A.* 104 14418–14423. 10.1073/pnas.0703126104 17726106 PMC1964825

[B197] Quintana-Cabrera R. Quirin C. Glytsou C. Corrado M. Urbani A. Pellattiero A. (2018). The cristae modulator Optic atrophy 1 requires mitochondrial ATP synthase oligomers to safeguard mitochondrial function. *Nat. Commun.* 9:3399. 10.1038/s41467-018-05655-x PMC6109181 30143614

[B198] Rafelski S. M. (2013). Mitochondrial network morphology: building an integrative, geometrical view. *BMC Biol.* 11:71. 10.1186/1741-7007-11-71 PMC3691739 23800141

[B199] Rai M. Katti P. Nongthomba U. (2014). Drosophila Erect wing (Ewg) controls mitochondrial fusion during muscle growth and maintenance by regulation of the Opa1-like gene. *J. Cell Sci.* 127 191–203. 10.1242/jcs.135525 24198395

[B200] Rambold A. S. Cohen S. Lippincott-Schwartz J. (2015). Fatty acid trafficking in starved cells: regulation by lipid droplet lipolysis, autophagy, and mitochondrial fusion dynamics. *Dev. Cell* 32 678–692. 10.1016/j.devcel.2015.01.029 25752962 PMC4375018

[B201] Rambold A. S. Kostelecky B. Elia N. Lippincott-Schwartz J. (2011). Tubular network formation protects mitochondria from autophagosomal degradation during nutrient starvation. *Proc. Natl. Acad. Sci. U.S.A.* 108 10190–10195. 10.1073/pnas.1107402108 21646527 PMC3121813

[B202] Ramos E. S. Motori E. Bruser C. Kuhl I. Yeroslaviz A. Ruzzenente B. (2019). Mitochondrial fusion is required for regulation of mitochondrial DNA replication. *PLoS Genet.* 15:e1008085. 10.1371/journal.pgen.1008085 31170154 PMC6553695

[B203] Rapizzi E. Pinton P. Szabadkai G. Wieckowski M. R. Vandecasteele G. Baird G. (2002). Recombinant expression of the voltage-dependent anion channel enhances the transfer of Ca2+ microdomains to mitochondria. *J. Cell Biol.* 159 613–624. 10.1083/jcb.200205091 12438411 PMC2173108

[B204] Rizzuto R. Brini M. Murgia M. Pozzan T. (1993). Microdomains with high Ca2+ close to IP3-sensitive channels that are sensed by neighboring mitochondria. *Science* 262 744–747. 10.1126/science.8235595 8235595

[B205] Rizzuto R. Pinton P. Carrington W. Fay F. S. Fogarty K. E. Lifshitz L. M. (1998). Close contacts with the endoplasmic reticulum as determinants of mitochondrial Ca2+ responses. *Science* 280 1763–1766. 10.1126/science.280.5370.1763 9624056

[B206] Rodriguez-Nuevo A. Diaz-Ramos A. Noguera E. Diaz-Saez F. Duran X. Munoz J. P. (2018). Mitochondrial DNA and TLR9 drive muscle inflammation upon Opa1 deficiency. *EMBO J.* 37:e96553. 10.15252/embj.201796553 PMC5978453 29632021

[B207] Romashko D. N. Marban E. O’Rourke B. (1998). Subcellular metabolic transients and mitochondrial redox waves in heart cells. *Proc. Natl. Acad. Sci. U.S.A.* 95 1618–1623. 10.1073/pnas.95.4.1618 9465065 PMC19119

[B208] Rossignol R. Gilkerson R. Aggeler R. Yamagata K. Remington S. J. Capaldi R. A. (2004). Energy substrate modulates mitochondrial structure and oxidative capacity in cancer cells. *Cancer Res.* 64 985–993. 10.1158/0008-5472.can-03-1101 14871829

[B209] Saxton W. M. Hollenbeck P. J. (2012). The axonal transport of mitochondria. *J. Cell Sci.* 125 2095–2104. 22619228 10.1242/jcs.053850 PMC3656622

[B210] Scalettar B. A. Abney J. R. Hackenbrock C. R. (1991). Dynamics, structure, and function are coupled in the mitochondrial matrix. *Proc. Natl. Acad. Sci. U.S.A.* 88 8057–8061. 10.1073/pnas.88.18.8057 1896451 PMC52445

[B211] Schauder C. M. Wu X. Saheki Y. Narayanaswamy P. Torta F. Wenk M. R. (2014). Structure of a lipid-bound extended synaptotagmin indicates a role in lipid transfer. *Nature* 510 552–555. 10.1038/nature13269 24847877 PMC4135724

[B212] Schorr S. van der Laan M. (2018). Integrative functions of the mitochondrial contact site and cristae organizing system. *Semin Cell Dev. Biol.* 76 191–200. 10.1016/j.semcdb.2017.09.021 28923515

[B213] Sesaki H. Jensen R. E. (1999). Division versus fusion: Dnm1p and Fzo1p antagonistically regulate mitochondrial shape. *J. Cell Biol.* 147 699–706. 10.1083/jcb.147.4.699 10562274 PMC2156171

[B214] Shaw C. S. Jones D. A. Wagenmakers A. J. (2008). Network distribution of mitochondria and lipid droplets in human muscle fibres. *Histochem. Cell Biol.* 129 65–72. 10.1007/s00418-007-0349-8 17938948

[B215] Sheftel A. D. Zhang A. S. Brown C. Shirihai O. S. Ponka P. (2007). Direct interorganellar transfer of iron from endosome to mitochondrion. *Blood* 110 125–132. 10.1182/blood-2007-01-068148 17376890

[B216] Shenouda S. M. Widlansky M. E. Chen K. Xu G. Holbrook M. Tabit C. E. (2011). Altered mitochondrial dynamics contributes to endothelial dysfunction in diabetes mellitus. *Circulation* 124 444–453. 10.1161/circulationaha.110.014506 21747057 PMC3149100

[B217] Shigenaga M. K. Hagen T. M. Ames B. N. (1994). Oxidative damage and mitochondrial decay in aging. *Proc. Natl. Acad. Sci. U.S.A.* 91 10771–10778. 10.1073/pnas.91.23.10771 7971961 PMC45108

[B218] Shirendeb U. P. Calkins M. J. Manczak M. Anekonda V. Dufour B. McBride J. L. (2012). Mutant huntingtin’s interaction with mitochondrial protein Drp1 impairs mitochondrial biogenesis and causes defective axonal transport and synaptic degeneration in Huntington’s disease. *Hum. Mol. Genet.* 21 406–420. 10.1093/hmg/ddr475 21997870 PMC3276281

[B219] Skulachev V. P. (1969). *Energy Accumulation in the Cell.* Moscow: Nauka Press.

[B220] Skulachev V. P. (1977). Transmembrane electrochemical H+-potential as a convertible energy source for the living cell. *FEBS Lett.* 74 1–9. 10.1016/0014-5793(77)80739-4 14031

[B221] Skulachev V. P. (1990). Power transmission along biological membranes. *J. Membr. Biol.* 114 97–112. 10.1007/bf01869092 2111408

[B222] Skulachev V. P. (2001). Mitochondrial filaments and clusters as intracellular power-transmitting cables. *Trends Biochem. Sci.* 26 23–29. 10.1016/s0968-0004(00)01735-7 11165513

[B223] Smirnova E. Goldberg E. B. Makarova K. S. Lin L. Brown W. J. Jackson C. L. (2006). ATGL has a key role in lipid droplet/adiposome degradation in mammalian cells. *EMBO Rep.* 7 106–113. 10.1038/sj.embor.7400559 16239926 PMC1369222

[B224] Smith H. E. Page E. (1977). Ultrastructural changes in rabbit heart mitochondria during the perinatal period: neonatal transition to aerobic metabolism. *Dev. Biol.* 57 109–117. 10.1016/0012-1606(77)90358-x 863101

[B225] Song M. Franco A. Fleischer J. A. Zhang L. Dorn G. W. II (2017). Abrogating mitochondrial dynamics in mouse hearts accelerates mitochondrial senescence. *Cell Metabolism* 26 872–883e5. 29107503 10.1016/j.cmet.2017.09.023 PMC5718956

[B226] Sood A. Jeyaraju D. V. Prudent J. Caron A. Lemieux P. McBride H. M. (2014). A Mitofusin-2–dependent inactivating cleavage of Opa1 links changes in mitochondria cristae and ER contacts in the postprandial liver. *Proc. Natl. Acad. Sci. U.S.A.* 111 16017–16022. 10.1073/pnas.1408061111 25352671 PMC4234614

[B227] Spinelli J. B. Haigis M. C. (2018). The multifaceted contributions of mitochondria to cellular metabolism. *Nat. Cell Biol.* 20 745–754. 10.1038/s41556-018-0124-1 29950572 PMC6541229

[B228] Stahon K. E. Bastian C. Griffith S. Kidd G. J. Brunet S. Baltan S. (2016). Age-related changes in axonal and mitochondrial ultrastructure and function in white matter. *J. Neurosci.* 36 9990–10001. 10.1523/jneurosci.1316-16.2016 27683897 PMC5039264

[B229] Stephan T. Roesch A. Riedel D. Jakobs S. (2019). Live-cell STED nanoscopy of mitochondrial cristae. *Sci. Rep.* 9:12419. 10.1038/s41598-019-48838-2 PMC6712041 31455826

[B230] Stoica R. De Vos K. J. Paillusson S. Mueller S. Sancho R. M. Lau K. F. (2014). ER-mitochondria associations are regulated by the VAPB-PTPIP51 interaction and are disrupted by ALS/FTD-associated TDP-43. *Nat. Commun.* 5:3996. 10.1038/ncomms4996 PMC4046113 24893131

[B231] Stowers R. S. Megeath L. J. Gorska-Andrzejak J. Meinertzhagen I. A. Schwarz T. L. (2002). Axonal transport of mitochondria to synapses depends on milton, a novel Drosophila protein. *Neuron* 36 1063–1077. 10.1016/s0896-6273(02)01094-2 12495622

[B232] Szabadkai G. Bianchi K. Varnai P. De Stefani D. Wieckowski M. R. Cavagna D. (2006). Chaperone-mediated coupling of endoplasmic reticulum and mitochondrial Ca2+ channels. *J. Cell Biol.* 175 901–911. 10.1083/jcb.200608073 17178908 PMC2064700

[B233] Szabadkai G. Simoni A. M. Chami M. Wieckowski M. R. Youle R. J. Rizzuto R. (2004). Drp-1-dependent division of the mitochondrial network blocks intraorganellar Ca2+ waves and protects against Ca2+-mediated apoptosis. *Mol. Cell* 16 59–68. 10.1016/j.molcel.2004.09.026 15469822

[B234] Tarnopolsky M. A. Rennie C. D. Robertshaw H. A. Fedak-Tarnopolsky S. N. Devries M. C. Hamadeh M. J. (2007). Influence of endurance exercise training and sex on intramyocellular lipid and mitochondrial ultrastructure, substrate use, and mitochondrial enzyme activity. *Am. J. Physiol. Regul. Integr. Comp. Physiol.* 292 R1271–R1278. 17095651 10.1152/ajpregu.00472.2006

[B235] Territo P. R. Mootha V. K. French S. A. Balaban R. S. (2000). Ca(2+) activation of heart mitochondrial oxidative phosphorylation: role of the F(0)/F(1)-ATPase. *Am. J. Physiol. Cell Physiol.* 278 C423–C435. 10666039 10.1152/ajpcell.2000.278.2.C423

[B236] Thivolet C. Vial G. Cassel R. Rieusset J. Madec A.-M. (2017). Reduction of endoplasmic reticulum-mitochondria interactions in beta cells from patients with type 2 diabetes. *PLoS One* 12:e0182027. 10.1371/journal.pone.0182027 28742858 PMC5526536

[B237] Tobias I. C. Khazaee R. Betts D. H. (2018). Analysis of mitochondrial dimensions and cristae structure in pluripotent stem cells using transmission electron microscopy. *Curr. Protoc. Stem. Cell Biol.* 47:e67. 10.1002/cpsc.67 30303625

[B238] Toulmay A. Prinz W. A. (2012). A conserved membrane-binding domain targets proteins to organelle contact sites. *J. Cell Sci.* 125 49–58. 10.1242/jcs.085118 22250200 PMC3269022

[B239] Tsushima K. Bugger H. Wende A. R. Soto J. Jenson G. A. Tor A. R. (2018). Mitochondrial reactive oxygen species in lipotoxic hearts induce post-translational modifications of AKAP121, DRP1, and OPA1 that promote mitochondrial fission. *Circ. Res.* 122 58–73. 10.1161/circresaha.117.311307 29092894 PMC5756120

[B240] Twig G. Elorza A. Molina A. J. Mohamed H. Wikstrom J. D. Walzer G. (2008a). Fission and selective fusion govern mitochondrial segregation and elimination by autophagy. *EMBO J.* 27 433–446. 10.1038/sj.emboj.7601963 18200046 PMC2234339

[B241] Twig G. Hyde B. Shirihai O. S. (2008b). Mitochondrial fusion, fission and autophagy as a quality control axis: the bioenergetic view. *Biochim. Biophys. Acta* 1777 1092–1097. 10.1016/j.bbabio.2008.05.001 18519024 PMC3809017

[B242] Valente A. J. Fonseca J. Moradi F. Foran G. Necakov A. Stuart J. A. (2019). Quantification of mitochondrial network characteristics in health and disease. *Adv. Exp. Med. Biol.* 1158 183–196. 10.1007/978-981-13-8367-0_10 31452141

[B243] Valm A. M. Cohen S. Legant W. R. Melunis J. Hershberg U. Wait E. (2017). Applying systems-level spectral imaging and analysis to reveal the organelle interactome. *Nature* 546 162–167. 10.1038/nature22369 28538724 PMC5536967

[B244] Vance J. E. (1990). Phospholipid synthesis in a membrane fraction associated with mitochondria. *J. Biol. Chem.* 265 7248–7256. 2332429

[B245] Vance J. E. Tasseva G. (2013). Formation and function of phosphatidylserine and phosphatidylethanolamine in mammalian cells. *Biochim. Biophys. Acta* 1831 543–554. 10.1016/j.bbalip.2012.08.016 22960354

[B246] Vernay A. Marchetti A. Sabra A. Jauslin T. N. Rosselin M. Scherer P. E. (2017). MitoNEET-dependent formation of intermitochondrial junctions. *Proc. Natl. Acad. Sci. U.S.A.* 114 8277–8282. 10.1073/pnas.1706643114 28716905 PMC5547643

[B247] Vincent A. E. Turnbull D. M. Eisner V. Hajnoczky G. Picard M. (2017). Mitochondrial nanotunnels. *Trends Cell Biol.* 27 787–799. 10.1016/j.tcb.2017.08.009 28935166 PMC5749270

[B248] Vincent A. E. White K. Davey T. Philips J. Ogden R. T. Lawless C. (2019). Quantitative 3D mapping of the human skeletal muscle mitochondrial network. *Cell reports* 26 996–1009.e4. 30655224 10.1016/j.celrep.2019.01.010 PMC6513570

[B249] Vogel F. Bornhövd C. Neupert W. Reichert A. S. (2006). Dynamic subcompartmentalization of the mitochondrial inner membrane. *J. Cell Biol.* 175 237–247. 10.1083/jcb.200605138 17043137 PMC2064565

[B250] Wang C. Taki M. Sato Y. Tamura Y. Yaginuma H. Okada Y. (2019). A photostable fluorescent marker for the superresolution live imaging of the dynamic structure of the mitochondrial cristae. *Proc. Natl. Acad. Sci. U.S.A.* 116 15817–15822. 10.1073/pnas.1905924116 31337683 PMC6689947

[B251] Wang H. Sreenivasan U. Hu H. Saladino A. Polster B. M. Lund L. M. (2011). Perilipin 5, a lipid droplet-associated protein, provides physical and metabolic linkage to mitochondria. *J. Lipid Res.* 52 2159–2168. 10.1194/jlr.m017939 21885430 PMC3220284

[B252] Wang X. Schwarz T. L. (2009). The mechanism of Ca2+ -dependent regulation of kinesin-mediated mitochondrial motility. *Cell* 136 163–174. 10.1016/j.cell.2008.11.046 19135897 PMC2768392

[B253] Weibel E. Kayar S. R. (1988). Matching O2 delivery to O2 demand in muscle: I. Adaptive variation. *Adv. Exp. Med. Biol.* 227 159–169. 10.1007/978-1-4684-5481-9_14 3289314

[B254] Westermann B. (2010). Mitochondrial fusion and fission in cell life and death. *Nat. Rev. Mol. Cell Biol.* 11 872–884. 10.1038/nrm3013 21102612

[B255] Westermann B. (2011). Organelle dynamics: ER embraces mitochondria for fission. *Curr. Biol.* 21 R922–R924. 22115460 10.1016/j.cub.2011.10.010

[B256] Westermann B. Prokisch H. (2002). Mitochondrial dynamics in filamentous fungi. *Fungal Genet. Biol.* 36 91–97. 10.1016/s1087-1845(02)00019-1 12081462

[B257] Wilkens V. Kohl W. Busch K. (2013). Restricted diffusion of OXPHOS complexes in dynamic mitochondria delays their exchange between cristae and engenders a transitory mosaic distribution. *J. Cell Sci.* 126 103–116. 10.1242/jcs.108852 23038773

[B258] Willingham T. B. Zhang Y. Andreoni A. Knutson J. R. Lee D. Y. Glancy B. (2019). MitoRACE: evaluating mitochondrial function in vivo and in single cells with subcellular resolution using multiphoton NADH autofluorescence. *J. Physiol.* 597 5411–5428. 10.1113/jp278611 31490555 PMC8366751

[B259] Wolf D. M. Segawa M. Kondadi A. K. Anand R. Bailey S. T. Reichert A. S. (2019). Individual cristae within the same mitochondrion display different membrane potentials and are functionally independent. *EMBO J.* 38:e101056. 10.15252/embj.2018101056 PMC6856616 31609012

[B260] Wong H. S. Dighe P. A. Mezera V. Monternier P. A. Brand M. D. (2017). Production of superoxide and hydrogen peroxide from specific mitochondrial sites under different bioenergetic conditions. *J. Biol. Chem.* 292 16804–16809. 10.1074/jbc.r117.789271 28842493 PMC5641882

[B261] Wong Y. C. Kim S. Peng W. Krainc D. (2019). Regulation and function of mitochondria-lysosome membrane contact sites in cellular homeostasis. *Trends Cell Biol.* 29 500–513. 10.1016/j.tcb.2019.02.004 30898429 PMC8475646

[B262] Wong Y. C. Ysselstein D. Krainc D. (2018). Mitochondria-lysosome contacts regulate mitochondrial fission via RAB7 GTP hydrolysis. *Nature* 554 382–386. 10.1038/nature25486 29364868 PMC6209448

[B263] Wu J. Cheng D. Liu L. Lv Z. Liu K. (2019). TBC1D15 affects glucose uptake by regulating GLUT4 translocation. *Gene* 683 210–215. 10.1016/j.gene.2018.10.025 30316925

[B264] Wu M. J. Chen Y. S. Kim M. R. Chang C. C. Gampala S. Zhang Y. (2019). Epithelial-Mesenchymal Transition Directs Stem Cell Polarity via Regulation of Mitofusin. *Cell Metab* 29 993–1002.e6. 30527740 10.1016/j.cmet.2018.11.004

[B265] Wust R. C. Grassi B. Hogan M. C. Howlett R. A. Gladden L. B. Rossiter H. B. (2011). Kinetic control of oxygen consumption during contractions in self-perfused skeletal muscle. *J. Physiol.* 589 3995–4009. 10.1113/jphysiol.2010.203422 21690197 PMC3179998

[B266] Yaffe M. P. (1999). Dynamic mitochondria. *Nat. Cell Biol.* 1 E149–E150. 10559975 10.1038/14101

[B267] Yamano K. Fogel A. I. Wang C. van der Bliek A. M. Youle R. J. (2014). Mitochondrial Rab GAPs govern autophagosome biogenesis during mitophagy. *Elife* 3:e01612. 10.7554/eLife.01612 PMC3930140 24569479

[B268] Yamano K. Wang C. Sarraf S. A. Munch C. Kikuchi R. Noda N. N. (2018). Endosomal Rab cycles regulate Parkin-mediated mitophagy. *Elife* 7:e31326. 10.7554/eLife.31326 PMC5780041 29360040

[B269] Yang C. Svitkina T. M. (2019). Ultrastructure and dynamics of the actin-myosin II cytoskeleton during mitochondrial fission. *Nat. Cell Biol.* 21 603–613. 10.1038/s41556-019-0313-6 30988424 PMC6499663

[B270] Yao C. H. Wang R. Wang Y. Kung C. P. Weber J. D. Patti G. J. (2019). Mitochondrial fusion supports increased oxidative phosphorylation during cell proliferation. *Elife* 8:e41351. 10.7554/eLife.41351 PMC6351101 30694178

[B271] Youle R. J. Karbowski M. (2005). Mitochondrial fission in apoptosis. *Nat. Rev. Mol. Cell Biol.* 6 657–663. 16025099 10.1038/nrm1697

[B272] Youle R. J. Narendra D. P. (2011). Mechanisms of mitophagy. *Nat. Rev. Mol. Cell Biol.* 12 9–14. 21179058 10.1038/nrm3028 PMC4780047

[B273] Youle R. J. van der Bliek A. M. (2012). Mitochondrial fission, fusion, and stress. *Science* 337 1062–1065. 10.1126/science.1219855 22936770 PMC4762028

[B274] Yu M. Nguyen N. D. Huang Y. Lin D. Fujimoto T. N. Molkentine J. M. (2019). Mitochondrial fusion exploits a therapeutic vulnerability of pancreatic cancer. *JCI Insight* 5:e126915. 10.1172/jci.insight.126915 PMC6777817 31335325

[B275] Yu R. Jin S. B. Lendahl U. Nister M. Zhao J. (2019). Human Fis1 regulates mitochondrial dynamics through inhibition of the fusion machinery. *EMBO J* 38:e99748. 10.15252/embj.201899748 PMC6463211 30842096

[B276] Yu S. B. Pekkurnaz G. (2018). Mechanisms Orchestrating mitochondrial dynamics for energy homeostasis. *J. Mol. Biol.* 430 3922–3941. 10.1016/j.jmb.2018.07.027 30089235 PMC6186503

[B277] Yu T. Robotham J. L. Yoon Y. (2006). Increased production of reactive oxygen species in hyperglycemic conditions requires dynamic change of mitochondrial morphology. *Proc. Natl. Acad. Sci. U.S.A.* 103 2653–2658. 10.1073/pnas.0511154103 16477035 PMC1413838

[B278] Zhang X. Cheng X. Yu L. Yang J. Calvo R. Patnaik S. (2016). MCOLN1 is a ROS sensor in lysosomes that regulates autophagy. *Nat. Commun.* 7:12109. 10.1038/ncomms12109 PMC4931332 27357649

[B279] Zhang X. M. Walsh B. Mitchell C. A. Rowe T. domain T. B. C. (2005). family, member 15 is a novel mammalian Rab GTPase-activating protein with substrate preference for Rab7. *Biochem. Biophys. Res. Commun.* 335 154–161. 10.1016/j.bbrc.2005.07.070 16055087

[B280] Zhao T. Huang X. Han L. Wang X. Cheng H. Zhao Y. (2012). Central role of mitofusin 2 in autophagosome-lysosome fusion in cardiomyocytes. *J. Biol. Chem.* 287 23615–23625. 10.1074/jbc.m112.379164 22619176 PMC3390636

[B281] Zhou Y. Carmona S. Muhammad A. Bell S. Landeros J. Vazquez M. (2019). Restoring mitofusin balance prevents axonal degeneration in a Charcot-Marie-Tooth type 2A model. *J. Clin. Invest.* 130 1756–1771. 10.1172/jci124194 30882371 PMC6436852

[B282] Zick M. Rabl R. Reichert A. S. (2009). Cristae formation—linking ultrastructure and function of mitochondria. *Biochimica. Biophys. Acta (BBA) Mol. Cell Res.* 1793 5–19. 10.1016/j.bbamcr.2008.06.013 18620004

